# Evolution of the Calcium-Based Intracellular Signaling System

**DOI:** 10.1093/gbe/evw139

**Published:** 2016-06-29

**Authors:** Elodie Marchadier, Matt E. Oates, Hai Fang, Philip C.J. Donoghue, Alistair M. Hetherington, Julian Gough

**Affiliations:** ^1^Life Sciences Building, University of Bristol, United Kingdom; ^2^GQE—Le Moulon, INRA, Univ. Paris-Sud, CNRS, AgroParisTech, Université Paris-Saclay, 91190 Gif-sur-Yvette, France; ^3^Department of Computer Sciences, University of Bristol, United Kingdom; ^4^Department of Earth Sciences, University of Bristol, United Kingdom

**Keywords:** protein architecture, calcium signaling, evolution, diversification, specialization

## Abstract

To progress our understanding of molecular evolution from a collection of well-studied genes toward the level of the cell, we must consider whole systems. Here, we reveal the evolution of an important intracellular signaling system. The calcium-signaling toolkit is made up of different multidomain proteins that have undergone duplication, recombination, sequence divergence, and selection. The picture of evolution, considering the repertoire of proteins in the toolkit of both extant organisms and ancestors, is radically different from that of other systems. In eukaryotes, the repertoire increased in both abundance and diversity at a far greater rate than general genomic expansion. We describe how calcium-based intracellular signaling evolution differs not only in rate but in nature, and how this correlates with the disparity of plants and animals.

## Introduction

Calcium is a ubiquitous intracellular second messenger in animals (Berridge et al. 2003) and plants (Dodd et al. 2010). In response to an extracellular stimulus the concentration of cytosolic free calcium ions increases from its resting level of around 100 nM to in the region of 1 µM. The increase in the concentration of cytosolic free calcium is typically fuelled by a combination of calcium influx through calcium-permeable channels and release of calcium from intracellular stores, such as the endoplasmic reticulum. This latter route typically involves the participation of other intermediary molecules (such as inositol,1,4,5 trisphosphate) that couple the perception of the extracellular stimulus (at the plasma membrane) to the intracellular stores. The intracellular environment contains a myriad of proteins that are able to bind nanomolar concentrations of calcium. Their properties change after binding calcium and these changes are responsible for coupling the increase in calcium (the calcium signal) to downstream reactions that culminate in the response to the primary stimulus. The cell also contains mechanisms to “switch-off” the calcium signal and these center on removing the calcium from the cytosol. The suite of proteins responsible for generating the intracellular calcium signal, responding to it and finally switching it off, have been termed the “calcium toolkit” by Berridge et al. (2003).

Although the eukaryotic calcium-based intracellular signaling system has been the subject of intense investigation for the past 40 years, we know rather less about calcium signaling in prokaryotes (Shemarova and Nesterov 2005b). However, the fact that it has been implicated in the control of cell division (Holland et al. 1999), chemotaxis (Tisa and Adler 1992), virulence, and biofilm formation (Sarkisova et al. 2005) suggests that calcium and more particularly calcium-based signaling is important in these organisms. More recently, studies demonstrate the involvement of calcium in cyanobacteria heterocyst differentiation (Hu et al. 2011) and bacterial cell wall biosynthesis (Nikolaidis et al. 2012), confirming the versatile function of this element in prokaryotes. Numerous prokaryotic calcium-binding proteins still remain to be functionally characterized to fully understand the role of this ion in bacteria (Michiels et al. 2002).

Given the central role of intracellular calcium signaling in the living world, a better understanding of the evolution of this calcium-signaling toolkit, and the proteins that comprise it, is crucial to our global understanding of cellular evolution. Some aspects of the evolution of calcium signaling have been the subject of recent reviews (Cai et al. 2015; Domínguez et al. 2015; Edel and Kudla 2015; Plattner 2015; Plattner and Verkhratsky 2015) that highlight the high conservation of the calcium toolkit from prokaryotes to metazoa and the increasing complexity of the proteins that make it up. The proteins that comprise the calcium-signaling toolkit are composed of modular domains. These domains, limited in number, are the evolutionary units producing (via duplication and recombination) the functionally diverse repertoire of proteins in a genome (Chothia and Gough 2009). An understanding of the evolution of the whole toolkit must take this into account. The Structural Classification of Proteins (SCOP) (Murzin et al. 1995) provides domains defined as evolutionary units and groups them into superfamilies whose members share a common evolutionary ancestor. The SUPERFAMILY resource (Gough et al. 2001; Gough and Chothia 2002) provides SCOP domain annotation of all completely sequenced genomes, including the evolutionary superfamily classification of individual domains and the domain architecture of each protein. Using the phylogenetic context of extant organisms, via a species tree of all completely sequenced genomes (de Lima Morais et al. 2011), we can reconstruct the domain architecture content of ancestral (extinct) eukaryote genomes. Most evolutionary studies of proteins examine the trajectory of a single family. Using established knowledge of calcium-based signaling pathways to identify actors in calcium-signaling, we can use the domain annotation (described above) to study the evolution of the toolkit as a whole.

The objective of our work is to investigate the evolution of the calcium-based signaling systems in order to account for the diversity that we see in extant organisms.

## Materials and Methods

### Identification of the Calcium-Binding Domains and Calcium-Signaling Components

Calcium-binding domains were identified by mining the literature and helped by taking advantage of keywords in the SUPERFAMILY, Gene Ontology (Ashburner et al. 2000) and Prosite (Sigrist et al. 2010) databases. They were manually curated to produce a collection constituting the 31 superfamilies presented in table 1.

**Table 1:** List of the 31 Calcium-Binding Domains with Their Correspondence to Prosite and the Link to Their Structure

Superfamily ID	Superfamily	Prosite Domain
47874	Annexin	PDOC00195
47473	EF-hand	PDOC50031;PDOC00018;PDOC00535
48619	Phospholipase A2, PLA2	PDOC00109
63446	Type I dockerin domain	PDOC00416
101887	Apyrase	
63829	Calcium-dependent phosphotriesterase	
49562	C2 domain (Calcium/lipid-binding domain, CaLB)	PDOC51210;PDOC00380
49899	Concanavalin A-like lectins/glucanases	PDOC51117;PDOC51328;PDOC00636;PDOC50092;PDOC50188
81653	Calcium ATPase, transduction domain A	PDOC00139
49313	Cadherin-like	PDOC00205
63887	P-domain of calnexin/calreticulin	PDOC00636
56784	HAD-like	PDOC00139
51735	NAD(P)-binding Rossmann-fold domains	PDOC51201;PDOC51201
56436	C-type lectin-like	PDOC00537
81660	Metal cation-transporting ATPase, ATP-binding domain N	PDOC00139
81665	Calcium ATPase, transmembrane domain M	PDOC00139
100895	Kazal-type serine protease inhibitors	PDOC00535
57196	EGF/Laminin	PDOC00913;PDOC51117;PDOC00535
57424	LDL receptor-like module	PDOC00929
103647	TSP type-3 repeat	PDOC51236;PDOC50092;PDOC51234
82895	TSP-1 type 1 repeat	PDOC50092
110083	Peptidylarginine deiminase Pad4, middle domain	
144270	Eferin C-derminal domain-like	
57630	GLA Gamma-carboxyglutamic acid-rich-domain	
48092	Transcription factor STAT-4 N-domain	
47668	N-terminal domain of cbl (N-cbl)	
101112	Oxygen-evolving enhancer protein 3,	
57581	TB module/8-cys domain	
103647	TSP type-3 repeat	
140570	MukF C-terminal domain-like	
82026	Calcium-mediated lectin	

To produce the functional labeling of full length protein domain architectures, the following procedure was followed. First calcium-signaling proteins were identified from: 1) KEGG (Ogata et al. 1999) pathways which include a calcium ion as a compound, the most important being the “Calcium-signaling pathway” (map04020); 2) a review by Kudla et al. (2010) describing calcium-signaling proteins; and 3) reviews by Shemarova and Nesterov (2005a, 2005b, 2007). The proteins identified were then grouped by component within the calcium-signaling toolkit. Using their UniProt (Apweiler et al. 2004) identifiers, the domain architectures of the proteins were extracted from the SUPERFAMILY database (see below). For components involving the ability to bind calcium, the architectures were restricted to those that include at least one of the 31 calcium-binding domains from table 1. All the functional component labels identified for the full length protein were inferred onto the participating calcium-binding architecture.

### Domain and Architecture Assignments in Genomes

The domain definitions and superfamily classification were taken from the SCOP database (Murzin et al. 1995). The protein sequences of the genomes used in this analysis, and the domain annotation of those proteins were taken from version 1.75 of the SUPERFAMILY database (de Lima Morais et al. 2011). The SUPERFAMILY domain annotation of genomes follows a well-established method, using a curated library of hidden Markov models incorporating an assignment procedure to generate protein domain architectures, described as a string of domains and their superfamily classifications. At the time this work was carried out the SUPERFAMILY database included the 1,558 distinct species used in this study, including 114 *Archaea*, 1,061 bacteria, and 383 eukayotes. Eukaryotes genomes include 163 fungi, 50 plants, and 116 *Metazoa* (among which 62 *Chordata*). From this taxonomy, 44 representative and model organisms were chosen in each super kingdoms for closer analysis in some parts of the work.

### Species Tree and Ancestral Genome Reconstruction

To carry out this work a species tree of all the completely sequenced genomes was required. The reference tree was downloaded from the SUPERFAMILY resource, where it was first described in Wilson et al. (2009) but the procedure has subsequently improved (Fang et al. 2013). In addition to the domain content of extant genomes (see above), the SUPERFAMILY resource also provides the molecular character content of ancestral genomes; this is limited to eukaryote lineages because of the prevalence of horizontal gene transfer in bacteria. The ancestral genome content from SUPERFAMILY allowed us to track the evolution of domain architectures in the calcium-signaling toolkit throughout eukaryote phylogeny. To facilitate the visualization of the ancestral genome contents and the evolutionary changes, we present these data phylogenetically. The boxes in figure 7 represent the common ancestors shared by the extant species from the most ancient (i.e., last eukaryote common ancestor [LECA], first line). The evolutionary history of a lineage can be traced by the corresponding column in this figure (from the top to the bottom).

### Limitations of the Analysis

The analysis presented here is dependent on our current knowledge of proteins, principally defined by: the domains of proteins in the PDB (Protein Data Bank) which have had their structures experimentally determined, the genomes that have been completely sequenced, and our ability to transfer the knowledge of one to the other via sequence homology. We only include such knowledge as exists and is encoded in the SUPERFAMILY resource, and thus our picture of the evolution of the calcium-based intracellular signaling system will not be complete. For example, the *Arabidopsis thaliana* vacuolar exchanger CAX proteins do not exhibit any SCOP domain annotation and are therefore uidentifiable in the genomes. However, roughly 70% of eukaryote proteins are annotated, and thus we expect that our fundamental findings will not be overturned by subsequent additions to the body of structural/genomic data. Since many of the proteins lacking annotation are likely to be intrinsically disordered, what we have presented covers a good majority of structured proteins; we anticipate that future work on unstructured proteins will have the most to add to this story.

## Results

### The Atypical Evolution of the Calcium-Signaling Toolkit

#### Several Different Families of Protein Structural Domains Bind Calcium, but Their Presence Is Not Uniform Across Species

**Fig. 1.**
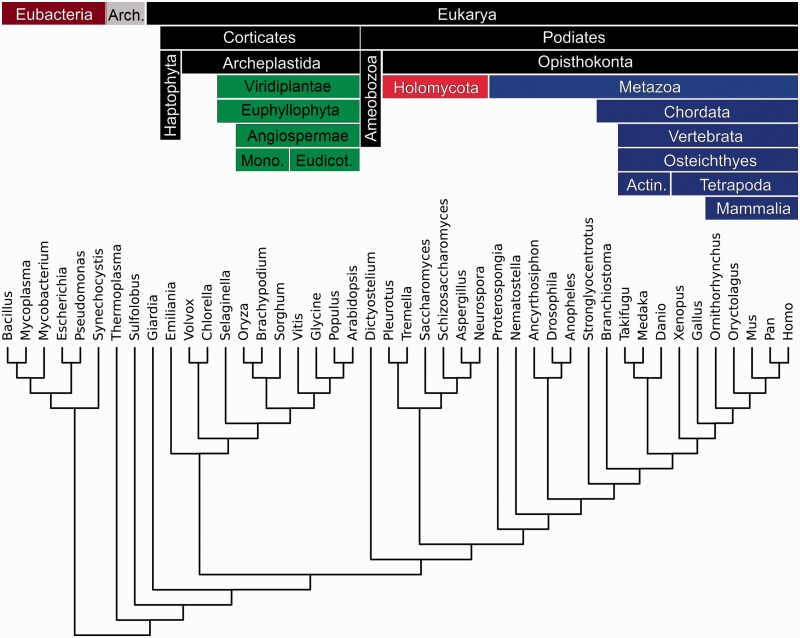
Phylogenetic tree of a subset of representative species of the diversity of organisms used in the study. Phylums are characterized by a color code that will be used in the next figures.

**Fig. 2.**
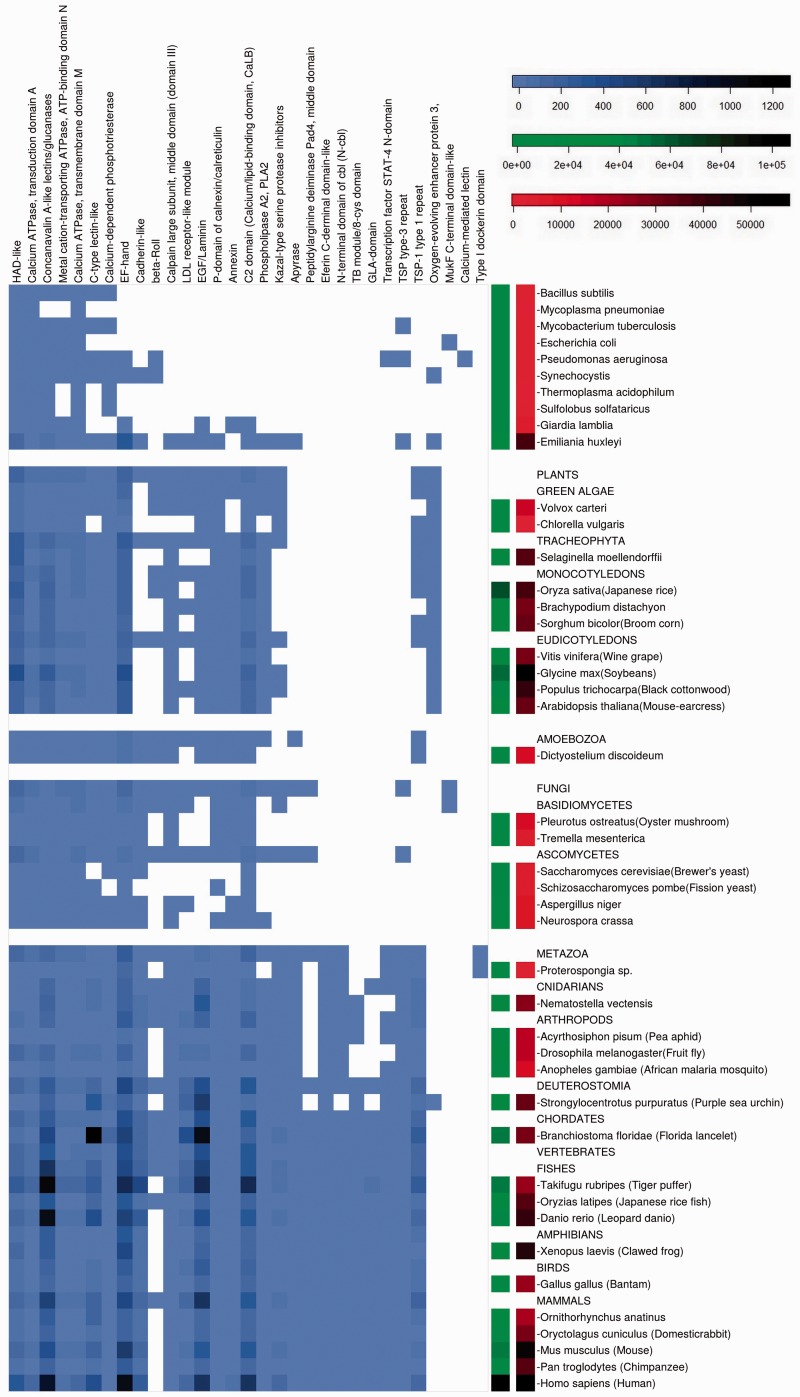
The occurences of building blocks of proteins (domains). Calcium-binding domains are labeled on the top horizontal axis. Both current and reconstructed ancestral organisms are labeled on the vertical axis. The entries of extint ancestral species represent their reconstructed proteome. The blue-scale of the matrix (on the left) stand for the number of proteins containing a calcium-binding domain that occur in each genome. The green-scale of the bar (middle) represents the diversity of proteins in the genome of each of the organisms (i.e., the size of the proteome). The red-scale of the bar (right side) represents the size of the genomes in number of genes. The scales of the color bar scale are indicated at the top right of the figure.

Calcium-binding domains are present in all organisms studied (fig.1), from simple prokaryotes to complex eukaryotes, but are heterogeneously distributed (fig. 2). On the whole the diversity increases with genome size, but there are also lineage-specific variations. The greatest diversity of calcium-binding domains is present in the higher eukaryotes and proteins containing these domains are also more abundant. This may either reflect important protein duplication events or the involvement of these domains in different protein architectures.

Under this first heading, we begin the results section by laying the context (below) for the rest of the results, by surveying these distributions of different domain superfamilies across species.

#### Proteins Containing Calcium-Binding Domains Have Diversified over Evolution by Domain Shuffling

**Fig. 3.**
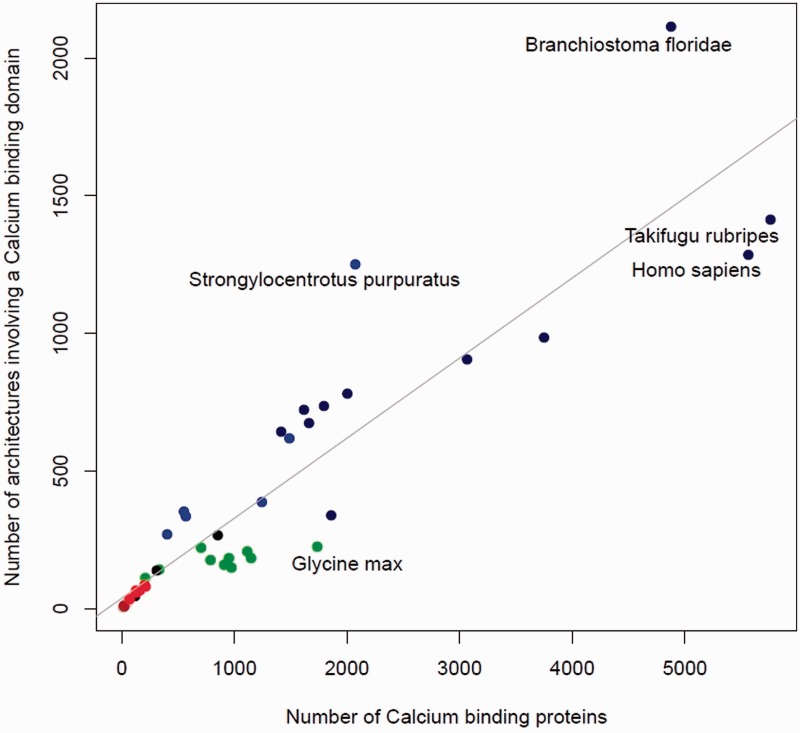
The number of domains correlating with the number of protein architectures. On the *y* axis is the number of different domain architectures containing at least one calcium-binding domain versus on the *x* axis the total number of proteins having these architectures. Each point on the graph represents a single proteome; the 44 proteomes used in figure 1 are plotted. *Archaea* are represented in brown, bacteria in gray, fungi in red, plants in green, chordates in dark blue, other metazoans in blue, and the rest of the eukaryotes in black. Shown in gray is a fitted regression line (Pearson correlation coefficient = 0.91).

From the previous section we see that in general more complex organisms have a greater number of calcium-binding domains, suggesting an increase in abundance during evolution. The relationship between the abundance and the diversity of proteins containing a calcium-binding domain is explored in figure 3. There is a high degree of correlation (Pearson correlation coefficient = 0.91) between the total number of proteins in a genome containing at least one calcium-binding domain, and the number of different domain architectures that describe them. The parallel increase in both protein abundance and diversity with organism complexity shows that the calcium-signaling toolkit has expanded over evolution not only by duplication but has been accompanied by an equal amount of recombination events (domain shuffling).

#### In Eukaryotes, Calcium-Binding Proteins Have Diversified More yet Been Duplicated Less than Other Proteins

**Fig. 4.**
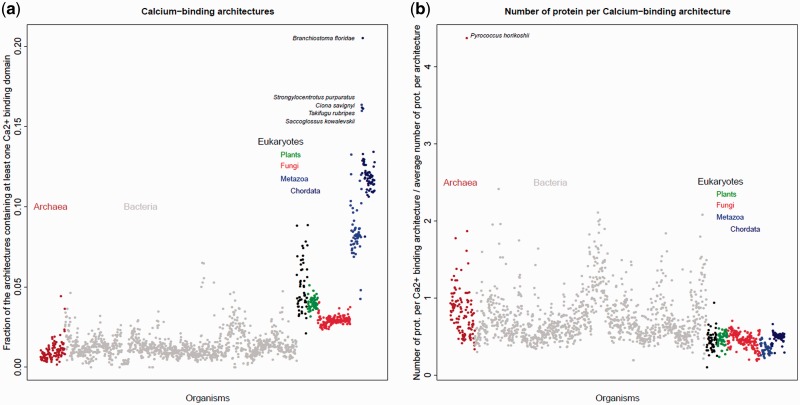
Left (*a*). Calcium-binding architectures diversity: fraction of architectures that contain at least one calcium-binding domain. Right (*b*). Calcium-binding architecture redundancy: the number of proteins per architecture expressed as a ratio between architectures containing a calcium-binding domain and all architectures in the proteome. On the *x* axis the 1,558 organisms are sorted by taxonomy. Each point represents a genome (colored by superkingdom).

The evolutionary trend linking expansion to some degree of diversification is shared by most proteins in the living world. In figure 4, we compare the evolution of the calcium-signaling toolkit to the rest of the proteome, and show it for all kingdoms of life. The diversity of calcium-binding proteins relative to others in the proteome varies little among prokaryotes. This relative diversity increases considerably in eukaryotes and even more in Metazoa and Chordata (fig. 4*a*), suggesting that calcium signaling contributed to the evolution of eukaryote complexity. Meanwhile, the number of proteins per architecture for calcium-binding proteins tends to be lower in eukaryotes than in bacteria and *Archaea* (fig. 4*a*). Thus in eukaryotes the calcium-signaling toolkit has evolved in a markedly different way to the rest of the proteome, and has been subject to much greater diversification than other pathways that have undergone expansion.

#### Some Architectures Are Dedicated to Calcium Signaling, While Others Play Multiple Roles

**Fig. 5.**
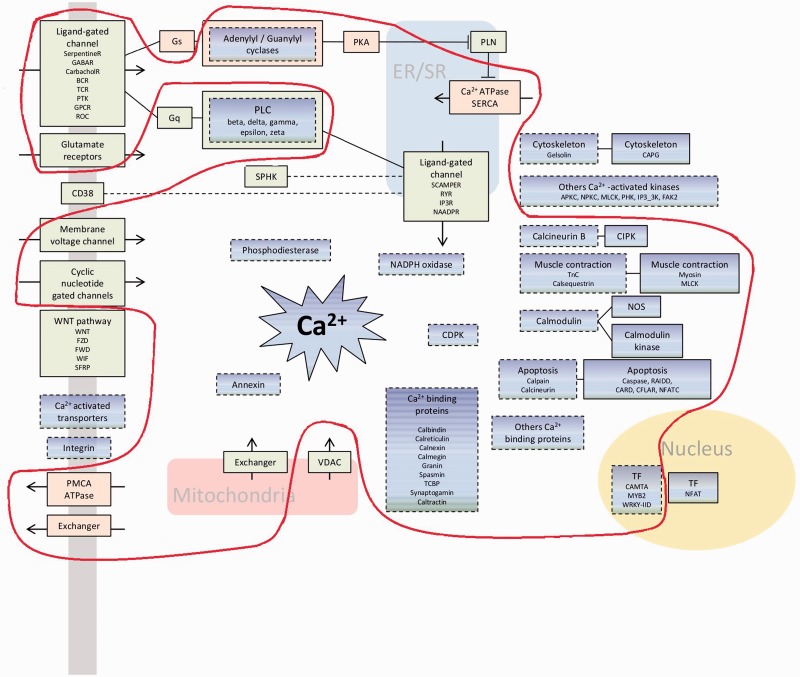
A schema of some components involved in calcium signaling. Boxes with dashed lines represent components able to bind calcium. Green boxes are calcium influx components, pink boxes are calcium efflux components, and blue boxes are signal decoding and relay components. The light red line encircles the minimal toolkit and includes boxes for which all organisms have at least one of the architecture of the component function.

**Fig. 6.**
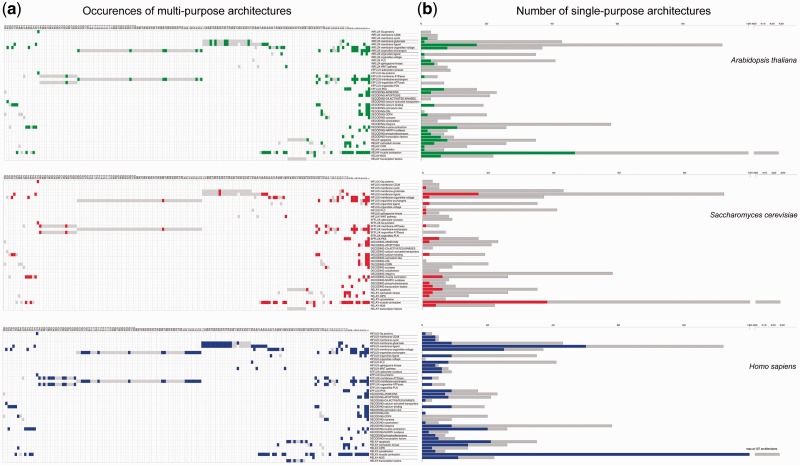
A functional breakdown of calcium-signaling architectures in three representative eukaryote genomes. The left part (*a*) shows multipurpose architectures and their functional components. Architectures present in the organism in question are displayed in colors, and totals for all organisms in gray. The right part (*b*) shows single-purpose architectures as histograms of the number (*y* axis) for each functional component (*x* axis). Data for the organism of interest are shown in colors, and totals for the 1,558 genomes are shown in gray.

The diversity of calcium-binding proteins that we observe can potentially confer many different calcium-dependent molecular functions in the living world. Calcium signaling requires the coordination of several components to generate, decode, and relay the calcium signal to the final effectors (fig. 5 and described in Plattner and Verkhratsky [2015]). Figure 5 shows that a breakdown of architectures by function reveals the existence of 23 architectures that enable the role of three or more different components of calcium signaling to be played by a single protein. Such highly multipurpose architectures are present in all ten chosen representative species including human, plants, and fungi (supplementary fig. S1, Supplementary Material online). Most proteins however only participate in one component, and histograms of their abundance are presented in figure 6. Looking across function, we observe that there is a greater proportion of single-purpose architectures for calcium signal decoding and relay, relative to multipurpose architectures.

In an attempt to define a minimal toolkit, in figure 5 we encircle those components which are in every single organism. This conserved core is well-defined since there are no components which would be included if we relaxed the criterion to only require a component to be in, for example, 90% of the genomes. On the other hand, the criterion for inclusion of a component in the conserved core of the toolkit is that every genome contains at least one architecture with a domain attributed with the function of that component. As a result of this, and due to some architectures having multiple purposes, a few components are included which are not likely to be present in all organisms; only the ancestral building-blocks are present in a different form. To illustrate by example, consider the muscle contraction component which contains the myosin domain, present in ATP-dependent motor proteins in human and used for muscle contraction: it is also present in proteins responsible for actin-based motility, which is present in all eukaryotic cells. A more detailed picture emerges below (table 2).

**Table 2:** Examples of Architectures Identified In the “Relay-Muscle Contraction” Component

Architectures Which Are Multi-Purpose and Present In Several Genomes	
47473	Homo sapiens: A2RRN2, calcyphosin Q02045, Myosin light chain 5 C9JV47, Grancalcin Arabidopsis thaliana: Q9LIK5, Calmodulin-like protein 11 Q9ZPX9, Calcium-binding protein KIC O81445, Calcineurin B-like protein 1 Colletotrichum graminicola: E3QMN8, Myosin regulatory light chain cdc4 Drosophila melanogaster: P42325, Neurocalcin homolog
Architectures Which Are Component-Specific (Single-Purpose) and Present in Several Genomes	
_gap_,56112	Homo sapiens: J3KQG8, Epithelial discoidin domain-containing recept O96013, Serine/threonine-protein kinase PAK 4 Arabidopsis thaliana: P0C5E2, Probable serine/threonine-protein kinase At1g18390 Q93V58, Serine/threonine-protein kinase GRIK1 C. elegans: P34314, Serine/threonine-protein kinase Saccharamyces cerevisiae: P36004, Probable serine/threonine-protein kinase KKQ8
Architectures Which Are Multipurpose and Genome-Specific (In Only One Genome)	
89837,89837,56112	Eimeria tenella: C8TE04, Myosin light chain kinase takes part of the “relay muscle contraction” and “relay calmodulin kinase” components
47473,_gap_,50729,56112,_gap_	Plasmodium yoelii yoelii: Q7RTG4, Myosin light chain kinase takes part of the “relay muscle contraction” and “decoding-CDPK” components
Architectures Which Are Component and Genome-Specific (Single-Purpose In Only One Genome)	
48726,48726,_gap_,48726,48726,48726,_gap_, 48726,48726,49265,56112,48726	Homo sapiens: D3DN97, Myosin light polypeptide kinase, isoform CRA_d
47473,55729	Harpegnathos saltator: E2BYA7, Myosin-2 essential light chain

The diversity of single-purpose architectures per component increases with organismal complexity (fig. 6), but none of the extant organisms’ genomes include all single-purpose architectures for any given component. This means that in addition to progressive growth of the shared calcium-signaling toolkit over eukaryote evolution, there are also lineage-specific additions in each species.

### Correlations between Organismal and Calcium-Signaling Evolution

**Fig. 7.**
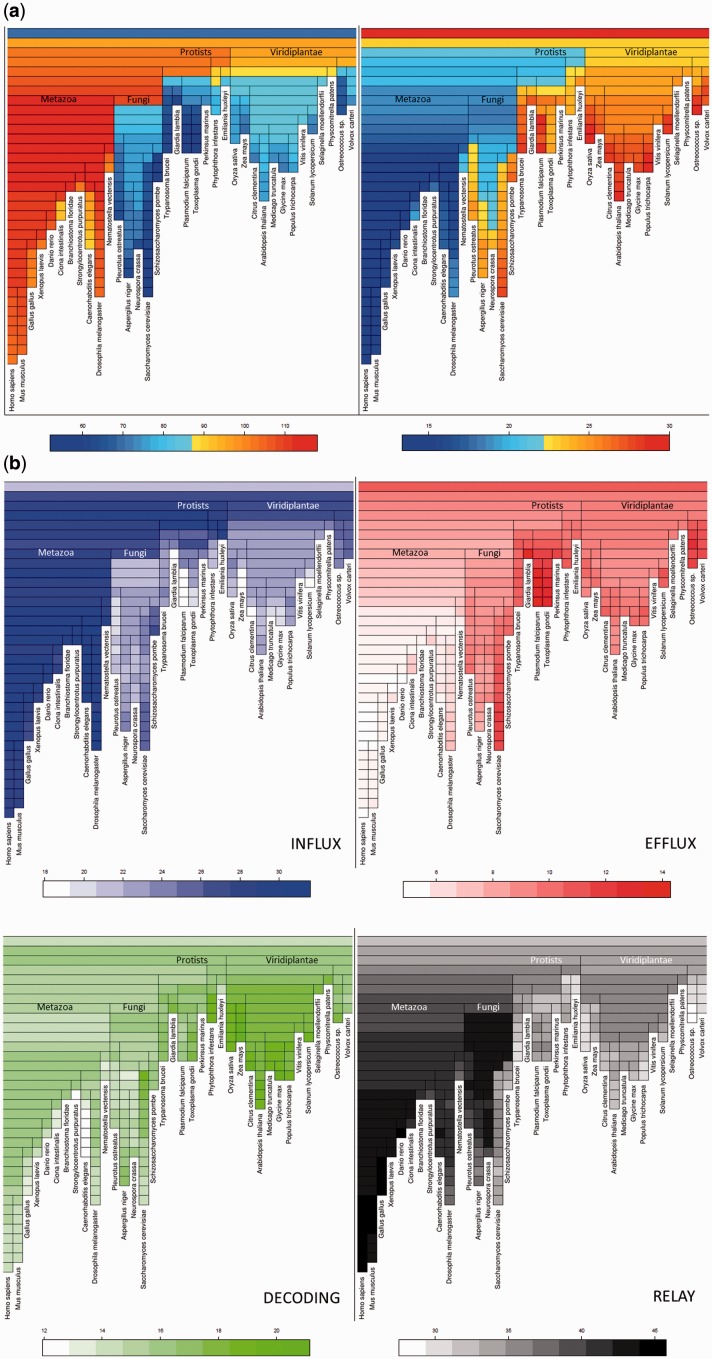
(*a*) Treemaps showing the reconstruction of the evolution of multipurpose architectures in calcium-signaling components from LECA to representative species of the eukaryotes. On the left, multipurpose architectures are expressed in numbers. On the right, multipurpose architectures are expressed in percentage of the architectures. (*b)* Treemaps showing the reconstruction of the evolution of the architecture diversity of calcium-signaling components from LECA to representative species of the eukaryotes*.* The percentages of the single purpose architectures dedicated to each component are indicated by the color-scale.

The creation and deletion of calcium-signaling domain architectures throughout evolution can be visualized using treemaps (fig. 7). The most striking observation is the global difference between multipurpose and single-purpose architectures, with multipurpose architectures surprisingly appearing earlier in eukaryote evolution. Other multipurpose architectures were continuously created all along the animal, fungal, and protist branches. Notably, the ancestral eukaryote had architectures for influx and efflux, afterwards influx was specifically expanded in animals (fig. 7*b*). Specialized architectures were created later than multipurpose ones and often in a phylum-specific manner, as shown for proteins involved in calcium efflux and calcium signal relay (fig. 7*b*).

#### Assembling LECA

A conserved core of calcium-binding superfamilies is present in all of the proteomes in the living world (Domínguez et al. 2015). Among these domains, the archetypal EF-hand calcium-binding motif and the calcium ATPase superfamilies are present in bacteria. Interestingly, some specific calcium-binding domains are also present in Prokaryotes. As an example, the “Oxygen-evolving enhancer protein 3” is represented in the proteome of the cyanobacteria *Synechocystis* which probably determines its ability to use photosynthesis (fig. 2).

There is very little variation in the diversity of calcium-binding proteins relative to the rest of the proteome among bacteria and archaea (fig. 4*a*). On the other hand, a lot of calcium-binding architectures are duplicated in these organisms (fig. 4*b*). For example, the archaebacterium *Pyrococcus horikoshii*, presents a surprisingly high duplication rate per architecture for calcium-signaling proteins (fig. 4*b*) which is explained by the high duplication rate of its genome (Kawarabayasi et al. 1998).

#### Last Eukaryote Common Ancestor

The recent study of the ciliated protozoan *Paramecium* showed that calcium signaling was already present in organisms at the unikonts–bikonts split (Plattner 2015). Our results showed the LECA was indeed potentially already able to generate and decode calcium signals as the domain architecture content of LECA included representatives from all of the main components of calcium signaling, including organelle specific Ca+-binding architectures from ancient endosymbiosis events (Blackstone 2015). Reconstruction of the domain architecture content of the LECA reveals the presence of representatives from all of the main components of calcium signaling (fig. 7*b*). This result is mirrored by the minimal toolkit defined in figure 5. Although LECA was potentially already able to generate and decode calcium signals, there were important reorganization events that redefined the calcium-signaling toolkits now seen in the living world.

#### “Protists”

Figures 2 and 4 demonstrate the high diversity of calcium-binding domains in unicellular eukaryotes (figs. 2 and 4*a*) and a strong specialization of their architectures (fig. 4b). Looking at protist species grouped together, we see an exaggerated variability in the diversity of their calcium toolkits. This can be explained by the heterogeneity of this paraphyletic grouping includes all eukaryotes except animals, plants, and fungi. In fact, protist genomes contain all of the calcium-binding domains that are found in plants and fungi (fig. 2).

As an example, the two thrombospondin domains (TSP type 1 and TSP type 3) have relatively different patterns of occurrence in the genomes (fig. 2). These repeats are found together in animals but more surprisingly in some (protist) stramenopile species, suggesting their joint presence in the ancient eukaryote ancestor. The type 3 domain was subsequently lost from the plant lineage and type 1 lost from the fungal lineage. Type 3 repeats are present also in bacteria which could correspond to the presence of an ancient form of the B type thrombospondin protein lacking type 1 repeat domains. The relative abundance of type 1 repeats is probably due to their involvement in diverse functions such as: angiogenesis inhibition (Iruela-Arispe et al. 1999), complement pathway (Patthy 1988), and apopotosis (Guo et al. 1997).

The chromalveolate (represented by the haptophyte *Emiliania huxleyi*) and plant lineages diverged more than 1500 Ma (Parfrey et al. 2011). Within the chromalveloate lineage, the calcium toolkit underwent a progressive increase, followed by a decline within the haptophyte lineage from 940 Ma which had a strong effect on the diversity of relay mechanisms manifest in *E. huxleyi*. A higher diversity of influx mechanisms is maintained than in the plants (represented by *A. thaliana*) during subsequent evolution. *Emiliania huxleyi* is one of the few protists possessing the oxygen-evolving enhancer domain which is commonly found in photosynthetic organisms (fig. 2). This domain was presumably acquired by plants from cyanobacteria during photosynthesis acquisition and this presence in the unicellular algae could be a key element in understanding how calcification affects photosynthesis in this organism (Gao et al. 2011).

#### Archaeplastida (Encompassing Green Algae and Plants)

The plant genomes present quite similar features in terms of calcium-binding domains and architectures (figs. 2 and 4). In addition, plants still have a substantial rate of duplication of their calcium-binding architectures (fig. 3). Despite a loss of multipurpose architectures along the course of plant evolution, the rate of multipurpose architectures in the calcium toolkit actually expanded at the cost of single purpose architectures (fig. 7*a*), contrary to the general trend.

However, the analysis of the evolution of single-purpose architectures in the calcium toolkit of plants reveals that their components evolved in different ways since LECA. Figure 7*b* demonstrates that green algae expanded their architectures dedicated to the generation of a calcium signal while they did not develop the decoding and relay mechanisms. On the contrary, in the rest of the plant lineage decoding and relay mechanisms are most extensively developed, including calmodulin and calmodulin-like proteins (Zhu et al. 2015). Subsequently, monocot diversification is characterized by a loss of relay-associated architectures.

Figure 7*b* shows that the plant lineage appears to have undergone compensatory changes in the mechanisms for calcium signal generation; species loosing some influx mechanisms expanded their efflux mechanisms (as in *Zea mays*, *Citrus clementina*, and *Solanum lycopersicum*), and vice versa (as in *Oryza sativa* and *A. thaliana*), probably to maintain their ability to generate a calcium signature. In plants, major gain and reorganization events appear to be initiated by decoding and relay components (at the beginning of plants’ speciation, just after protists’ divergence) which suggests that the requirement of new calcium signatures leads to them being strongly selected for.

#### Opisthokonta (Divergence of Animals and Fungi)

The major eukaryotic event after the split from plants was the speciation of fungi and animals. From this time onwards, components able to decode and relay calcium signals expanded their diversity of architectures. Figure 7 shows the increase over animal evolution of the number of calcium toolkit genes for the influx, decoding, relay, and efflux mechanisms since the split with fungi. An example of an expansion in the later stage of an effector pathway is the calcineurin B-like (CBL) and CBL-interacting protein kinases (CIPK) domains (supplementary fig. S2, Supplementary Material online), which are, respectively, able to sense a calcium signal and relay the information, functioning together (Edel and Kudla 2015). During evolution, the number of CIPK architectures increased more than for the CBL proteins (supplementary fig. S2, Supplementary Material online). Another example is with calcium modulated “calmodulins” which are able to regulate different biological processes in eukaryotes by activating calmodulin-dependent kinases. Proteins with calmodulin-like architectures are conserved from early eukaryotes in almost all species, as are calmodulin-dependant kinase proteins, with the latter having expanded in animals (and to a lesser extent plants) (supplementary fig. S2, Supplementary Material online). These specific observations are indicative of a widely seen trend, which makes sense in terms of cellular cost, since a greater expansion of the late effectors saves on the number of parallel pathways created.

#### Holomycota (Fungi)

Numerous fungal genomes are available, and loosely speaking these species have calcium-binding domains and architectures that are similar to those found in plants, except those related to photosynthesis (figs. 2 and 4*a*). However, the repertoire of their calcium-binding domains contains a few unusual features such as the presence of the eukaryote-specific Pad4 (protein arginine deiminase 4) protein middle domain, which is generally considered to be a vertebrate-specific enzyme (Bachand 2007). Another one is the MukF protein superfamily, thought to function exclusively in bacterial chromosome segregation; we find that it is also present in Basidiomycetes highlighting the need to investigate their role in these fungi. In general, the speciation of the fungal lineage started with a decrease in the calcium toolkit which can be explained by a substantial loss of multipurpose architectures (fig. 7*a*). However, this event correlates with the reorganization of functions achieved by single purpose architectures (fig. 7*b*). Notably, an expansion of relay mechanisms is observed simultaneously with a decrease of influx proteins. This is followed by species-specific creation and loss events corresponding to the signal generation mechanisms (fig. 7*b*). Interestingly, *Saccharomyces* and *Schizosaccharomyces* lineages expanded their efflux mechanisms independently more than other ascomycetes, and consequently lost a part of the relay system. This probably reflects the existence of a more direct action of calcium in these organisms.

#### Metazoa (Animals)

There is a considerable increase in the relative diversity of calcium-binding architectures associated with the lineage leading to metazoa after it separated from Holomycota (fig. 4). Despite the existence of numerous multipurpose architectures in the first Metazoa, the subsequent speciation events saw a decline in the importance of these proteins in the calcium toolkit (fig. 7*a*), meaning that specialized architectures (single purpose) were created during the evolution of the animal lineage. Figure 7*b* shows the timeline of the evolution of the major functions from LECA to the human lineage. The divergence of plants and fungi is followed by the loss of many protein domain architectures able to provoke the entrance of calcium into the cell, while conversely they were maintained in the Metazoa. In contrast, the part of the calcium toolkit that is dedicated to the release of calcium out of the cell, decreased in the animal lineage.

An increase in calcium decoding architectures is associated with Bilateria. It demonstrates that the eumetazoan ancestor had a substantial calcium-signaling toolkit at the point when cnidarians diverged. For example, some current cnidarians and arthropods have a TB module/8-cys domain, characteristic of the TGF-beta binding protein, which is mainly present in chordates (fig. 2). In addition, the GLA-domain Gamma-carboxyglutamic acid-rich is predominantly found in chordates where it has been implicated in blood coagulation and bone mineralization (Morita et al. 1984) (via coagulation factors and osteocalcin), however it is also present in conotoxin, a secreted toxin in marine gastropods and in Cnidarians such as the coral *Acropora digitifera.*

#### Chordata

Extensive reorganization, including losses of decoding architectures and gains of relay mechanisms, are associated with the origin of chordates. The calcium-signaling toolkit of the chordates reached the highest diversity in the cephalochordates lineage (fig. 4*a*). This observation is consistent with the existence of a calmodulin multigene family in *Branchiostoma floridae* that Karabinos and Bhattacharya (2000) describe as having evolved independently to the vertebrate calmodulin family. Such an independent evolution could have profound implications for the amphioxus calcium-signaling toolkit, especially those proteins containing C-type lectin domains.

#### Mammals

Figure 7 shows that there continued up to the present-day human to be a few losses of architectures related to calcium influx and calcium signal decoding, mostly in the 200 Myr after the speciation of mammals (the cells of which already had an abundant calcium-signaling toolkit). Most of the single purpose architectures of the mammalian calcium toolkit are involved in calcium influx and signal relay (fig. 7*a*). Despite their late speciation, Mammals are far from containing the whole diversity of the calcium toolkit (fig. 6).

Interestingly, the Beta-Roll structure appears in a wide range of species including some chordates but has been lost in most mammals (fig. 2). This domain commonly found in serralysins has initially been identified in an alkaline protease from *Pseudomonas aeruginosa* (Baumann et al. 1993) but its presence has previously been suggested in the platyhelminthe *Echinococcus granulosus* (Rodrigues et al. 1997). Our data for eukaryotes indicate that this domain is much more widespread in the living world than currently assumed.

In the same way that an increased diversity of influx and efflux proteins in animals allowed the generation of more complex signals, we might expect that more organelle-specific components could lead to more calcium signatures via control of cellular stores of calcium ions that can be released into the cytoplasm. Curiously the evolution of organelle-specific architectures does not seem to correlate well with the evolution of intracellular organelles. Some influx organelle-specific architectures were present in ancient eukaryotes, undergoing an increase in diversity during major steps of evolution of the vertebrates (supplementary fig. S2, Supplementary Material online). This reveals that organelles became an important player in the calcium-signaling toolkit of vertebrates thanks to new calcium-binding architectures, while plants, unicellular protists, and fungi lacked such specific structures. Contrary to what is seen in plants, in Metazoa it appears that the calcium generation mechanisms were already in place when the decoding and relay mechanisms expanded.

## Discussion

Since the first computational sequence alignments became possible, there have been a great number of evolutionary studies of gene families, but to progress our understanding of molecular biology closer to the level of the cell, we must consider the evolution of whole systems. The calcium**-**signaling system is a key component of many essential functions in plants, animals, and bacteria with implications for multicellularity and the evolution of complexity in higher organisms. We have described the calcium**-**signaling toolkit in terms of the proteins that can be found in the genomes of cellular organisms, the conserved structural domains which comprise them, and the types of roles that their individual functions play within calcium signaling (see “Limitations of the Analysis” in Materials and Methods section). We have shown that the molecular components of the toolkit in each organism, from the most complex downwards, are not just smaller and smaller subsets of each other. The LECA had a surprisingly extensive toolkit, and we have shown that several calcium-signaling domains (e.g., the Beta-Roll structure) are more widespread in nature than previously thought. As well as revealing promising clues to understanding fungal and protist biology, the presence in ancestral species of domains that are relevant to the biology of higher organisms (e.g., GLA), suggests that functional studies of them in a simpler organism may be possible. Since the eukaryote ancestor there have been numerous lineage-specific variations along the dividing evolutionary paths, leading to significant differences in the calcium-signaling protein repertoire in the genomes of extant organisms. In general, eukaryotes have expanded their repertoire of proteins over time both in number and variation via domain duplication and recombination (Chothia and Gough 2009). We present strong evidence that the calcium-signaling toolkit underwent an expansion in eukaryotes at a far greater rate than other functions, and with an increased rate of diversification that coincides with prior hypotheses of increasing organismal complexity as measured by cell type diversity (Vogel and Chothia 2006).

Examining the different functional components of the calcium-signaling toolkit, we reveal that calcium signature generation and signature decoding are both ancient cellular properties. In eukaryotes, we observe that multipurpose proteins (able to carry out more than one function) were present in the ancestor, but that as organisms increased in complexity, they did so by increasing the abundance and diversity of single-purpose proteins. This direction of evolution might be counterintuitive in terms of complexity, and is the opposite to the general trend of gene fusion being more common than gene fission (Kummerfeld and Teichmann 2005). The evolution of calcium signaling is an exceptional case relative to the general trend of proteome evolution. Gene fusion can be a mechanism for increasing efficiency within a pathway by combining components. In contrast, our data suggest that the calcium-signaling toolkit evolved to increase the number of different signatures that can be communicated and was not driven by efficiency. Single-purpose proteins (with selection acting independently on them), allow a more efficient expansion of signal transmission paths than multipurpose proteins. This is because, as we observe, by evolving progressively more proteins at later stages of the pathway, the number of redundant parallel pathways required to deliver the number of signals is reduced. This suggests that calcium signaling may operate more like a decision tree than a collection of independent linear pathways. This decision tree may look like a bow tie where the calcium ion is in the center with inputs and outputs fanning out either side (Dodd et al. 2010).

To better understand calcium signaling we turn to a comparison of the reconstructed human and plant lineages. The evolution of the toolkit in animals and plants appears to be substantially different, with plants showing a greater degree of coevolution between the major functions and animals expanding mechanisms for decoding and signal relay rather than creating new mechanisms for generating signatures. This abundance and diversity of calcium-signaling proteins coincided with the diversification of complex animals (Fernandez-Busquets 2010; Verkhratsky and Parpura 2014), during which the toolkit consolidated under selection. In the plant lineage there is an independent example of environmental calcium concentration affecting the size of the toolkit during evolution; we observe a transient increase in protein diversity when *Chlorophyta* and *Streptophyta* diverged in freshwater and seawater habitats (Becker and Marin 2009) leading to a subsequent loss of diversity in decoding and relay mechanisms of *Streptophyta* (fig. 7)

In conclusion, we have investigated aspects of calcium-signaling evolution in eukaryotes, taking into consideration the genomic repertoire of proteins and their domains, the functional components of the toolkit and environmental factors. We have discovered that the evolution of calcium signaling is fundamentally different to other protein evolution in general. Through this exemplar study of an important signaling system we have shown that the popular approach of studying protein families should be extended to whole systems, a key step toward understanding the evolution of organisms at the cellular, anatomical, and ultimately whole-organism levels.

## Supplementary Material

Supplementary figures S1 and S2 are available at *Genome Biology and Evolution* online (http://www.gbe.oxfordjournals.org/).

Supplementary Data
