# Supplementary material for: Evolution of the Calcium-Based Intracellular Signaling System
Source: Genome Biol Evol. 2016 Jun 29;8(7):2118–32. doi: 10.1093/gbe/evw139 (PMC4987107; doi:10.1093/gbe/evw139)
Supplement: Supplementary Data [file supp_8_7_2118__index.html]

Evolution of the Calcium-Based Intracellular Signaling System — Supplementary Data 

# Evolution of the Calcium-Based Intracellular Signaling System

## Supplementary Data

files

- Supplementary Data - jpg file
- Supplementary Data - pdf file
