# Supplementary figures and images for: Evolution of the Calcium-Based Intracellular Signaling System
Source: Genome Biol Evol. 2016 Jun 29;8(7):2118–32. doi: 10.1093/gbe/evw139 (PMC4987107; doi:10.1093/gbe/evw139)

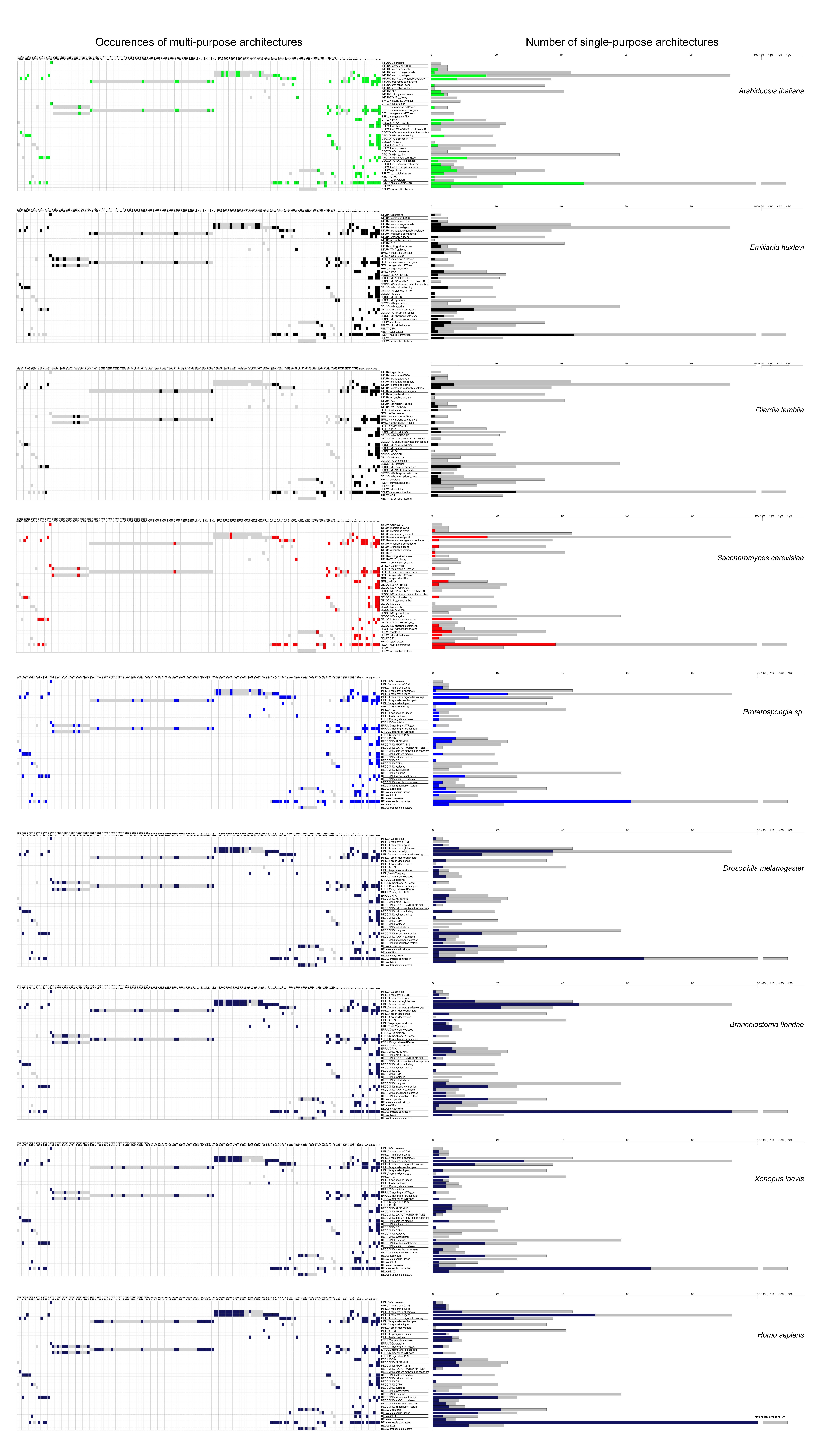

Supplement: Supplementary Data [file supp_evw139_FigureS1.jpg]
