# Supplementary material for: Evolution of the Calcium-Based Intracellular Signaling System
Source: Genome Biol Evol. 2016 Jun 29;8(7):2118–32. doi: 10.1093/gbe/evw139 (PMC4987107; doi:10.1093/gbe/evw139)
Supplement: Supplementary Data [file supp_evw139_FigureS2.pdf]

Influx

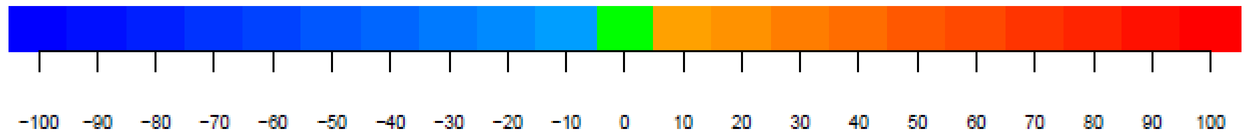

Color scale = Percentage of the total number of architectures in a component that have been lost or gained

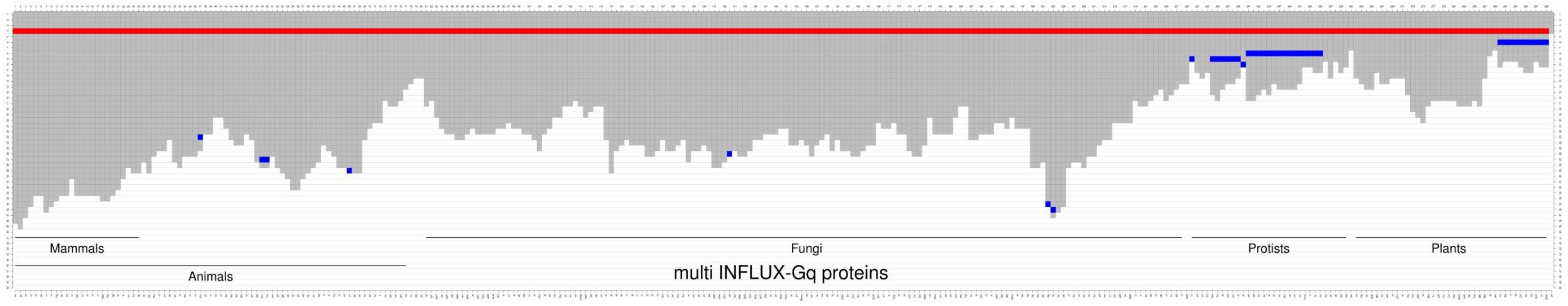

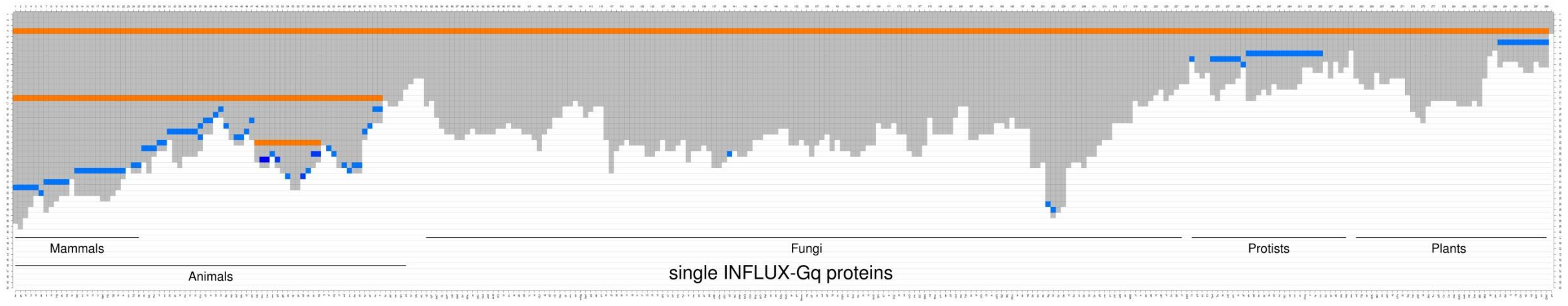

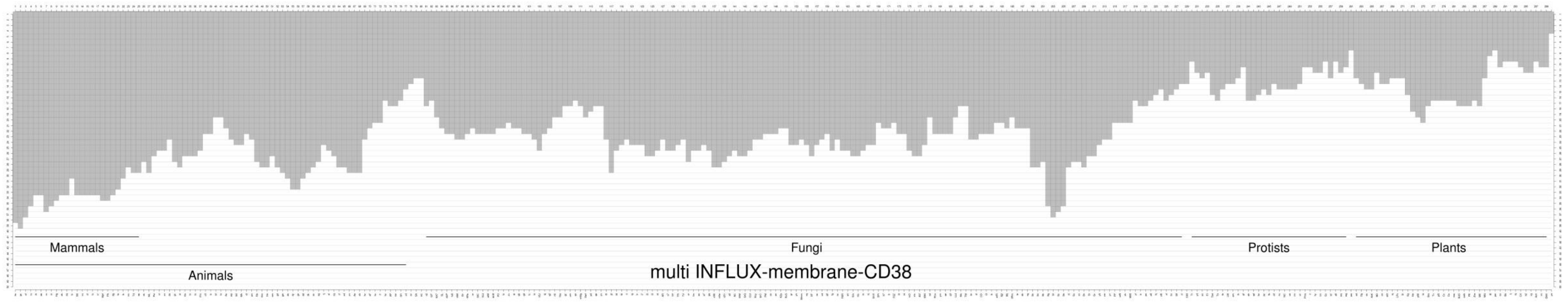

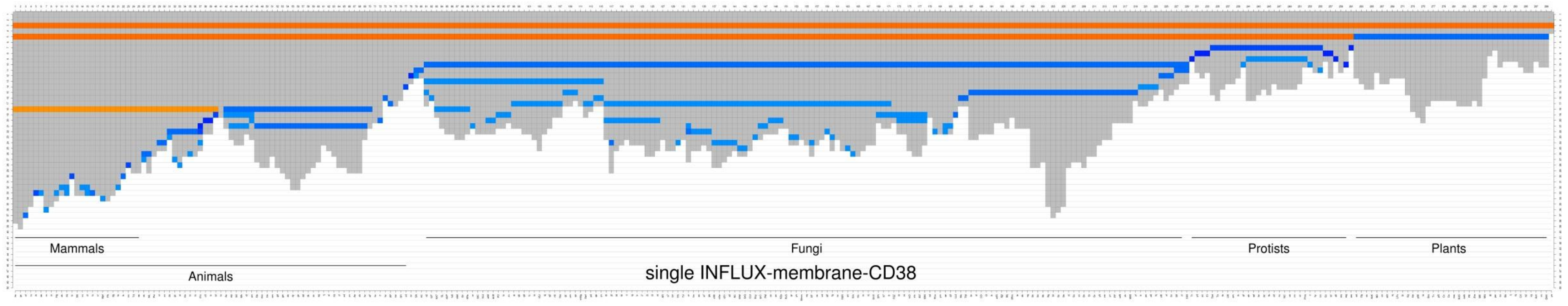

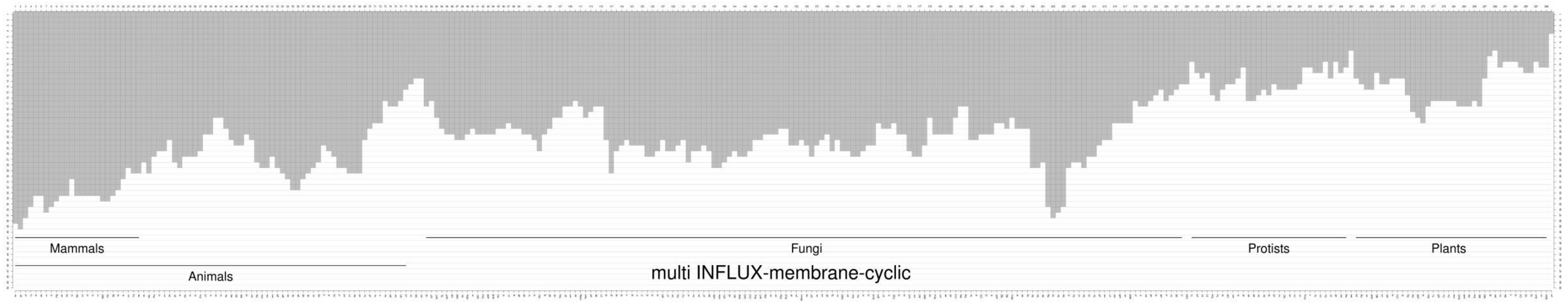

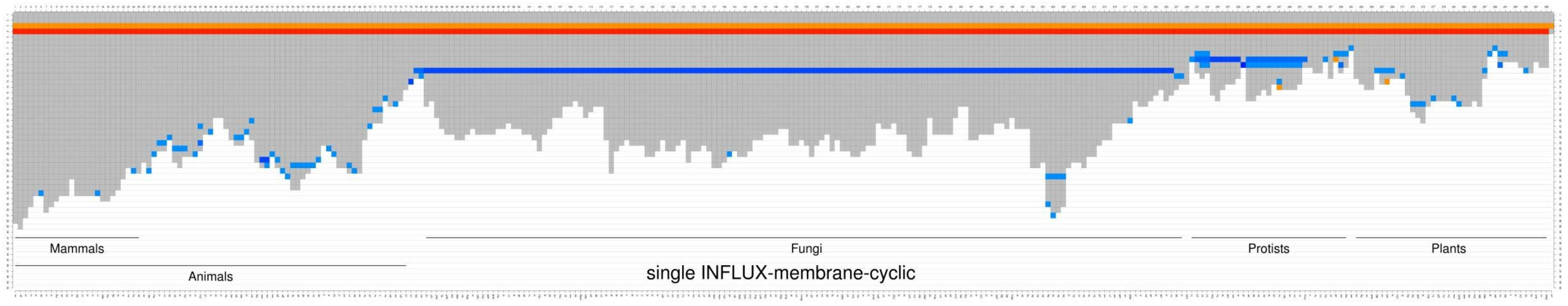

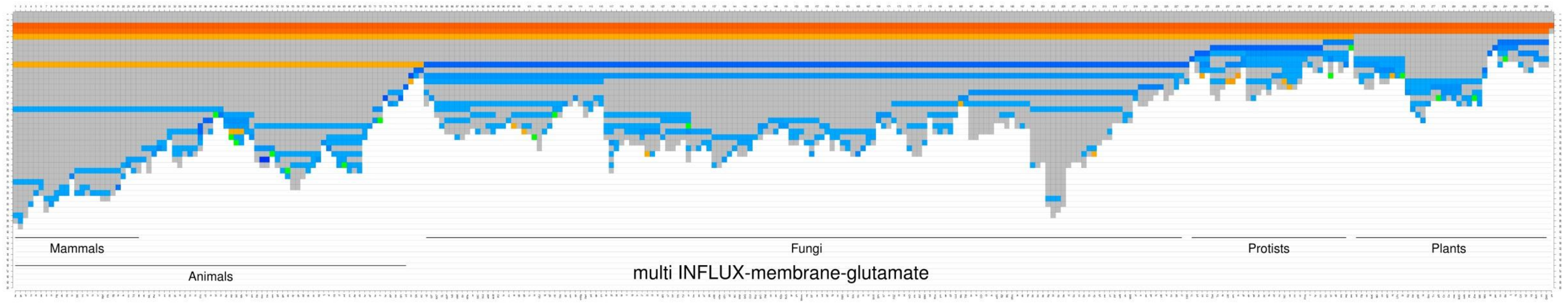

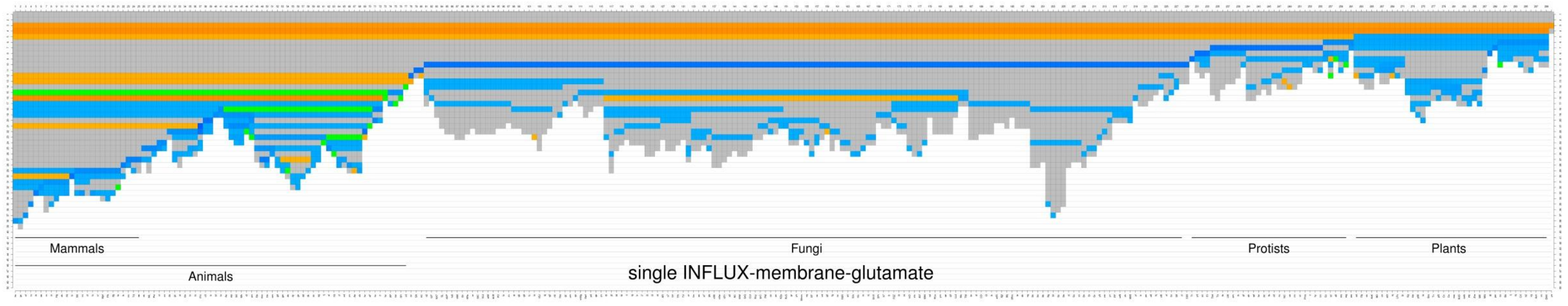

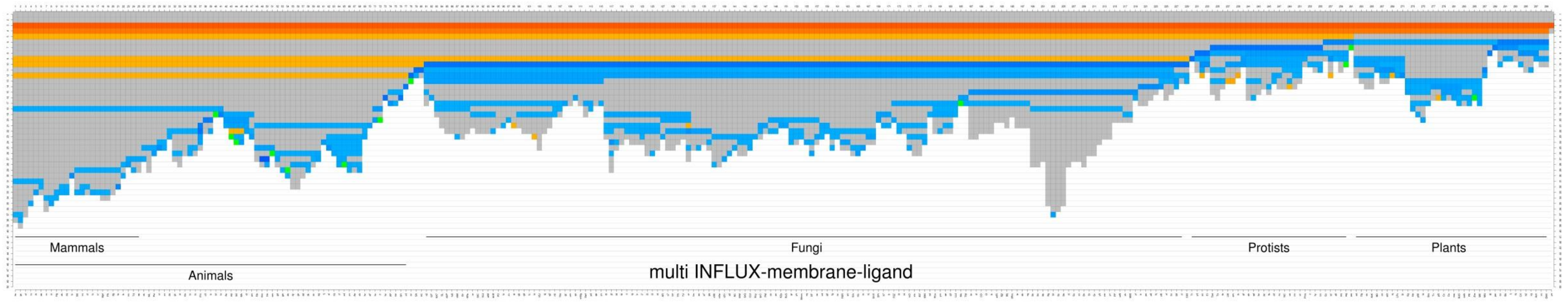

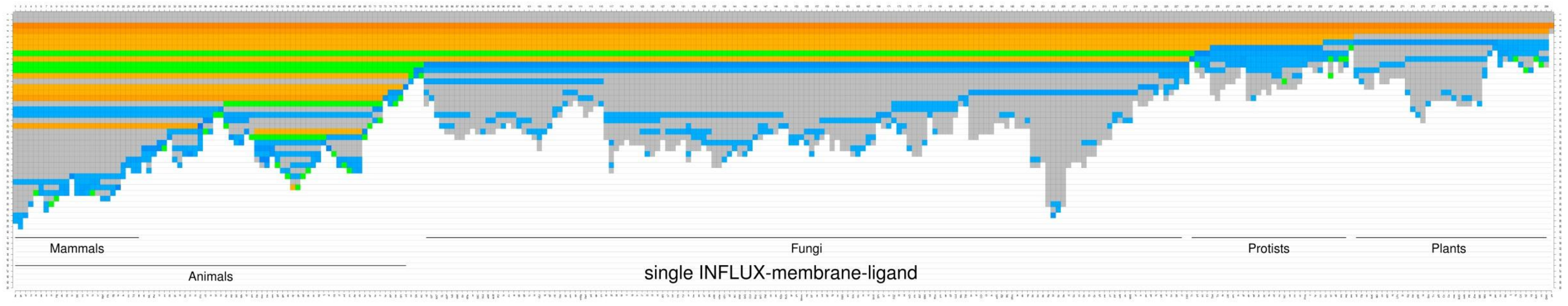

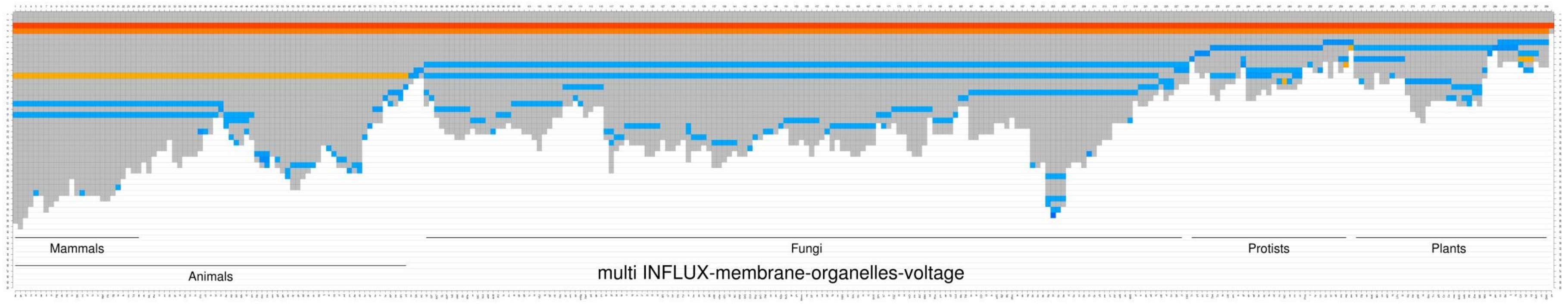

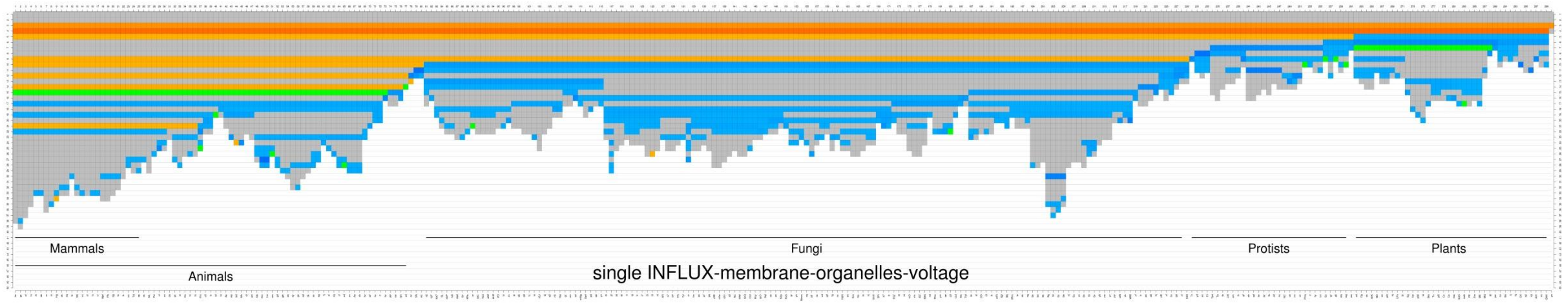

Influx

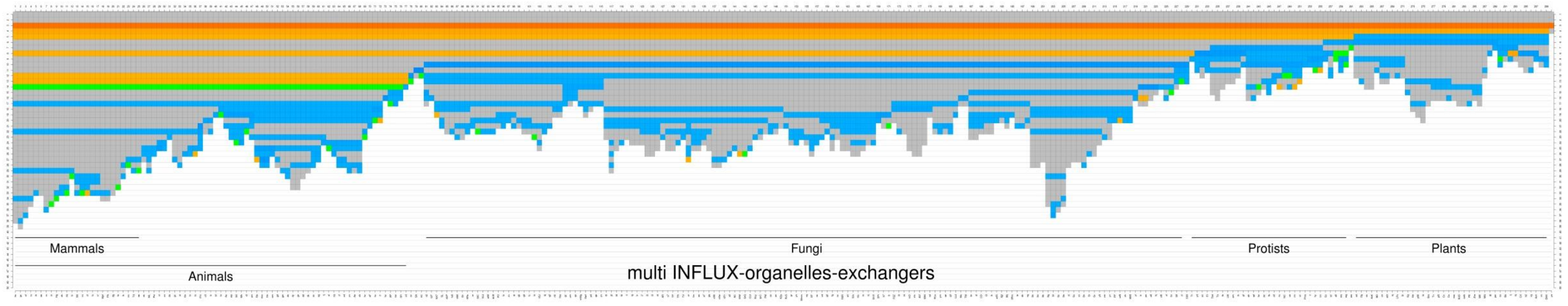

multi INFLUX-organelles-exchangers

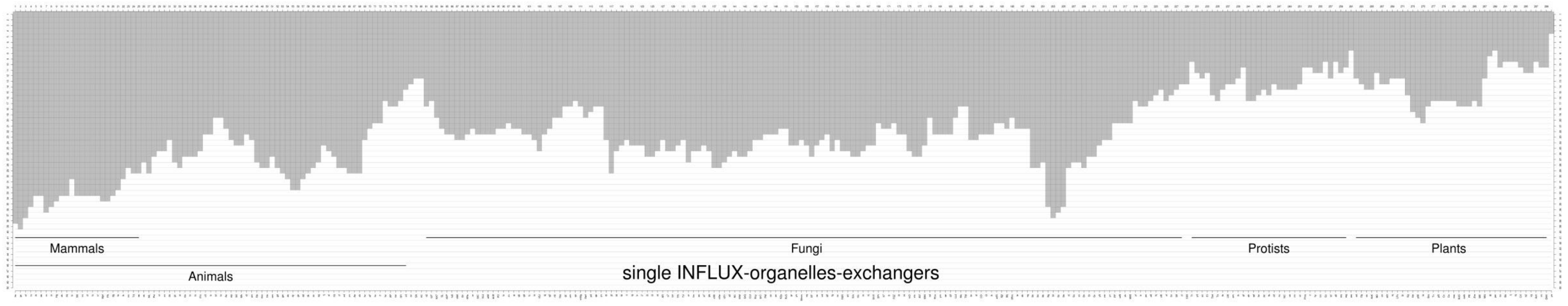

single INFLUX-organelles-exchangers

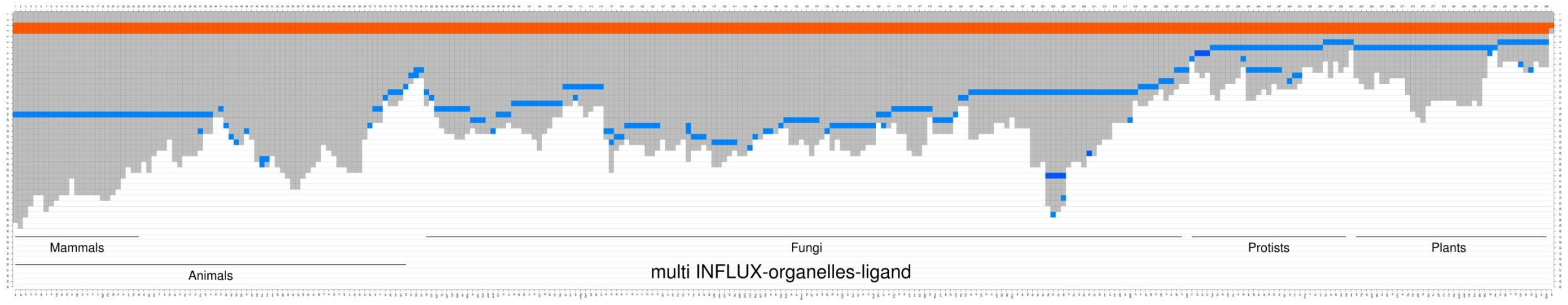

multi INFLUX-organelles-ligand

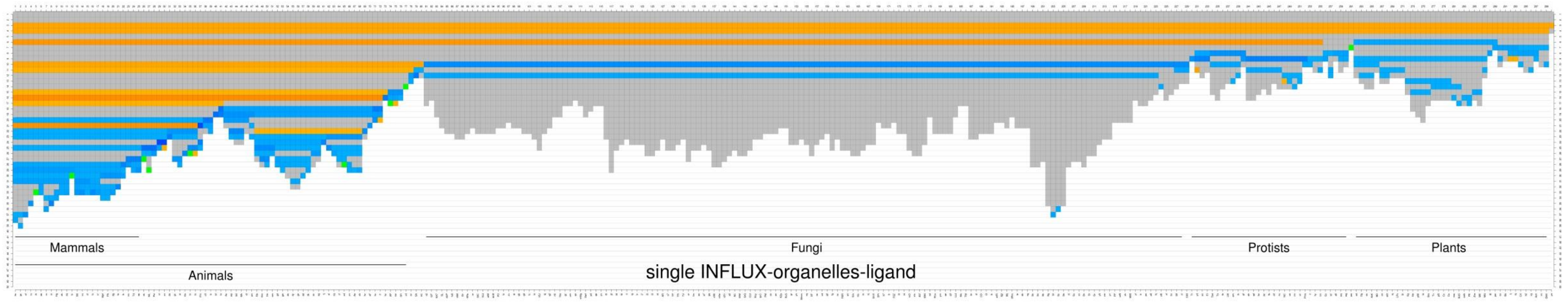

single INFLUX-organelles-ligand

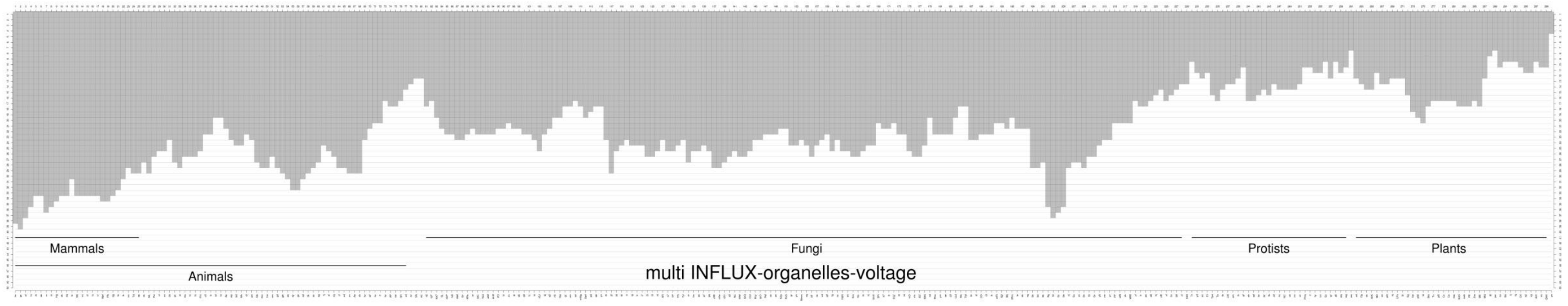

multi INFLUX-organelles-voltage

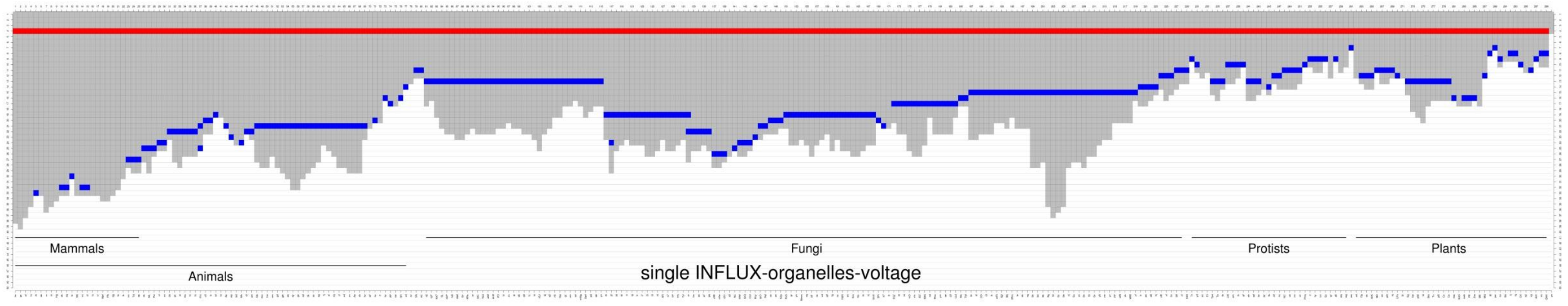

single INFLUX-organelles-voltage

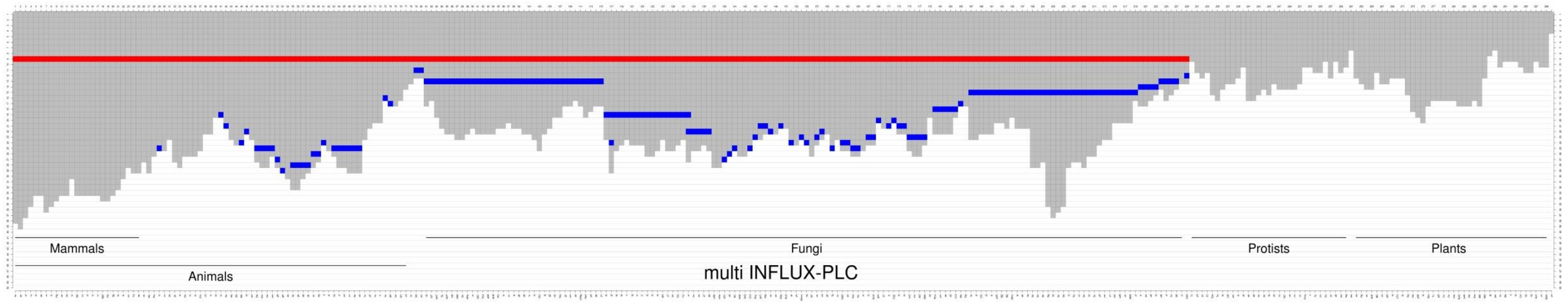

multi INFLUX-PLC

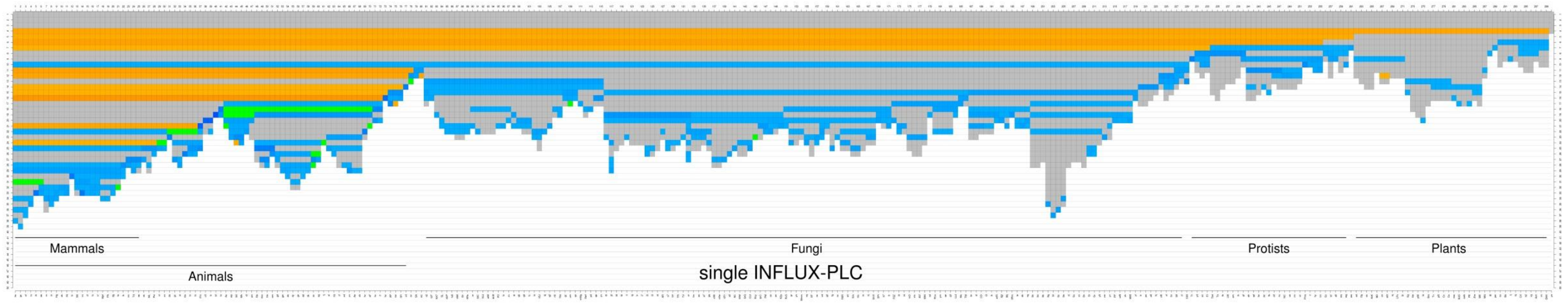

single INFLUX-PLC

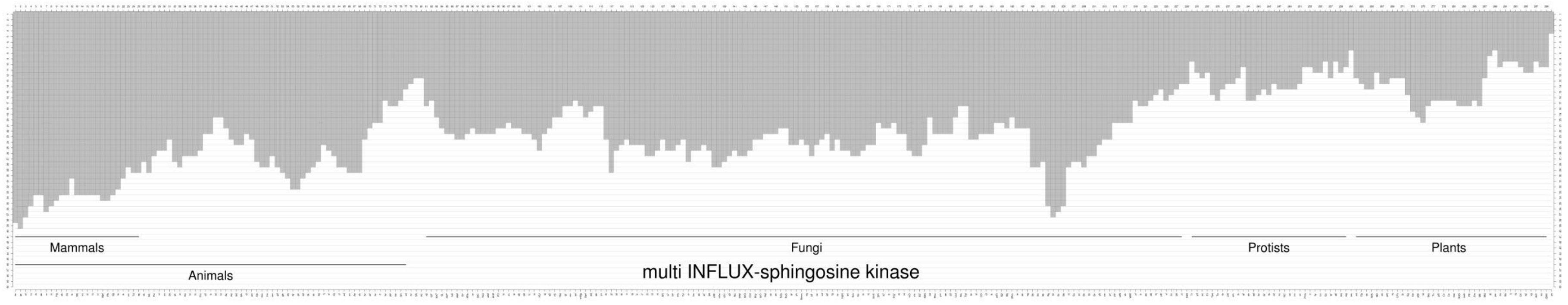

multi INFLUX-sphingosine kinase

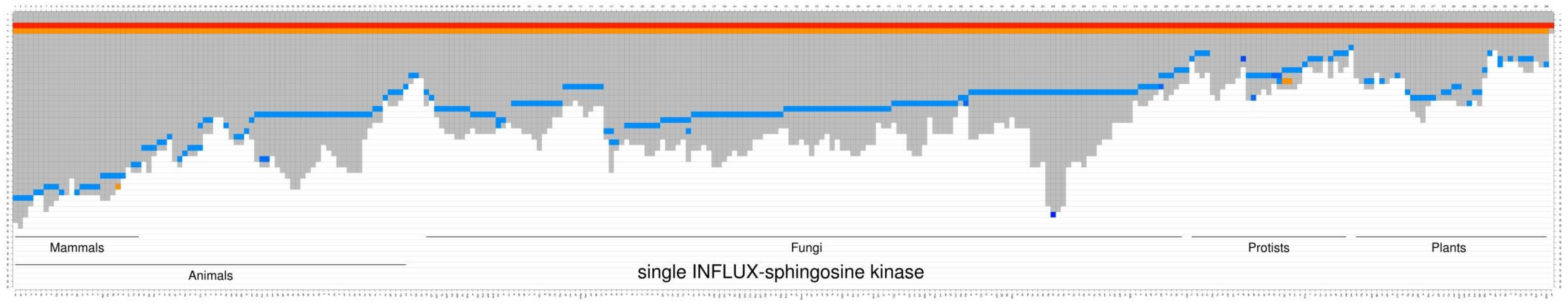

single INFLUX-sphingosine kinase

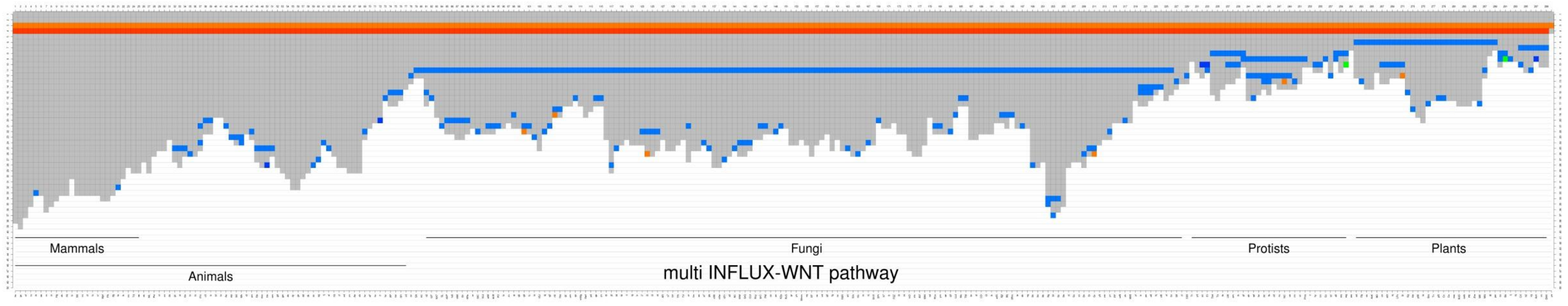

multi INFLUX-WNT pathway

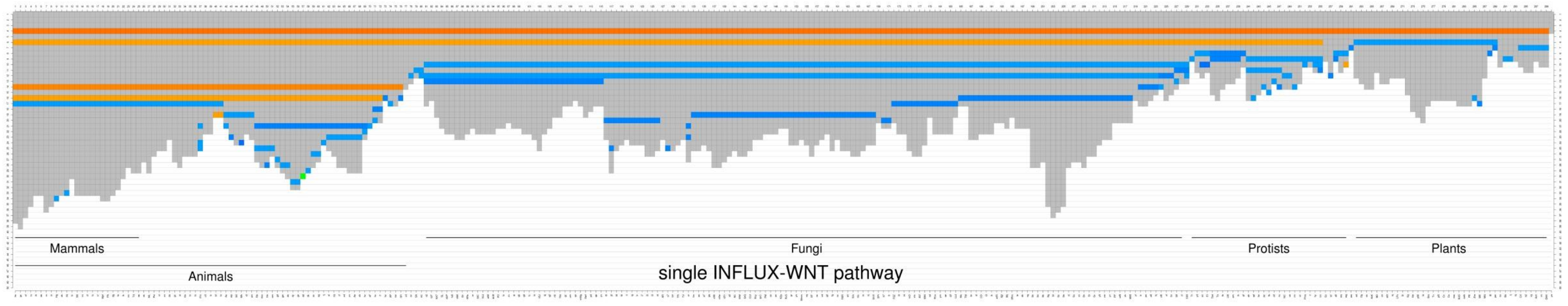

single INFLUX-WNT pathway

Efflux

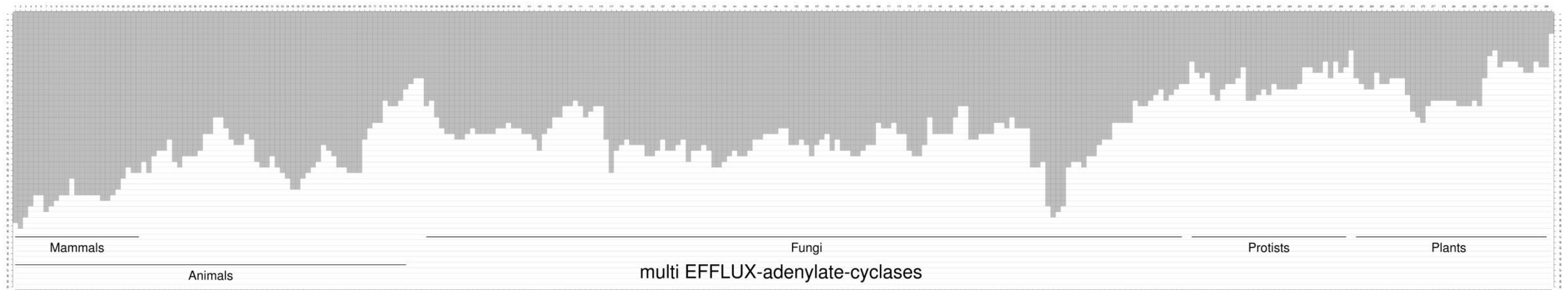

multi EFFLUX-adenylate-cyclases

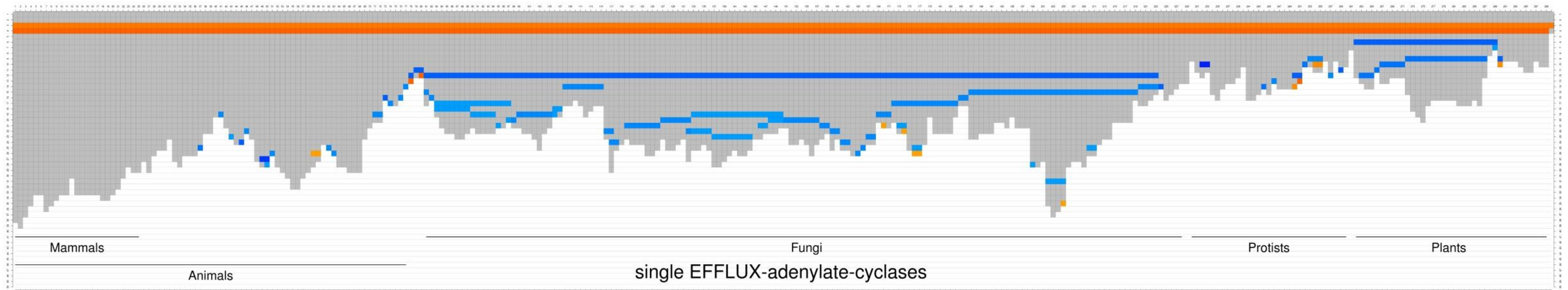

single EFFLUX-adenylate-cyclases

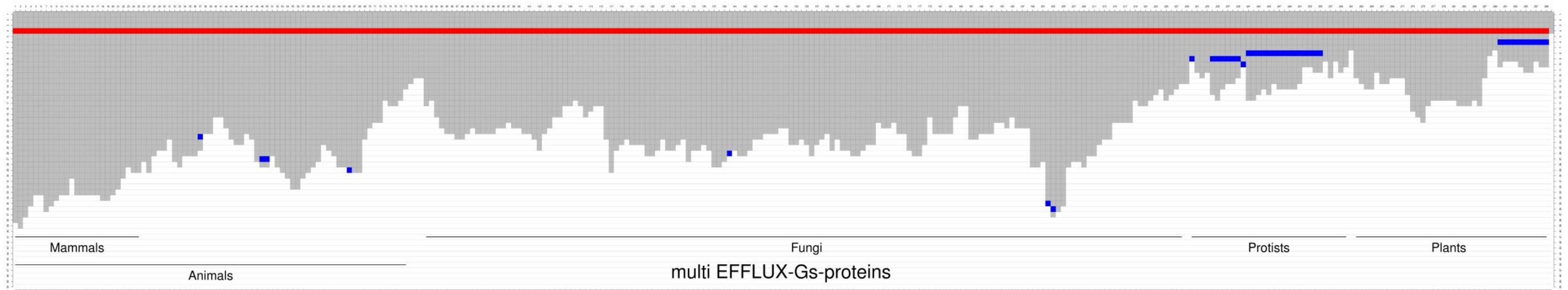

multi EFFLUX-Gs-proteins

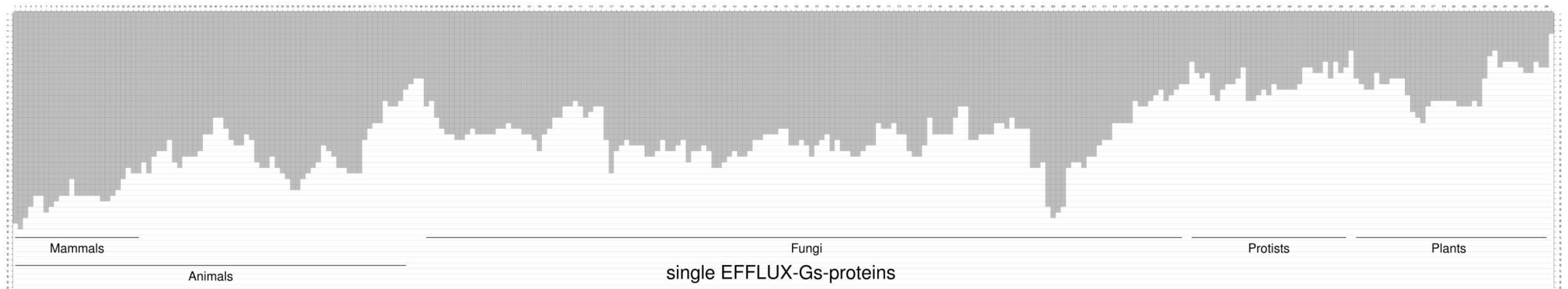

single EFFLUX-Gs-proteins

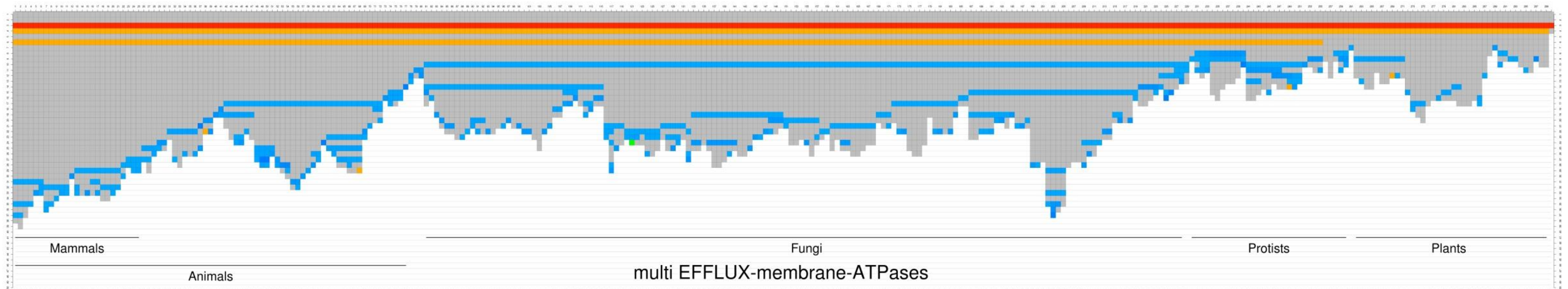

multi EFFLUX-membrane-ATPases

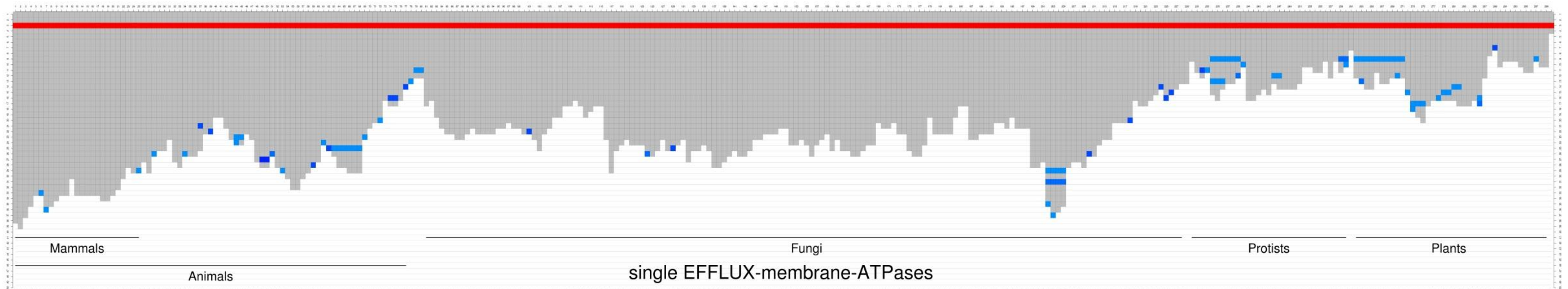

single EFFLUX-membrane-ATPases

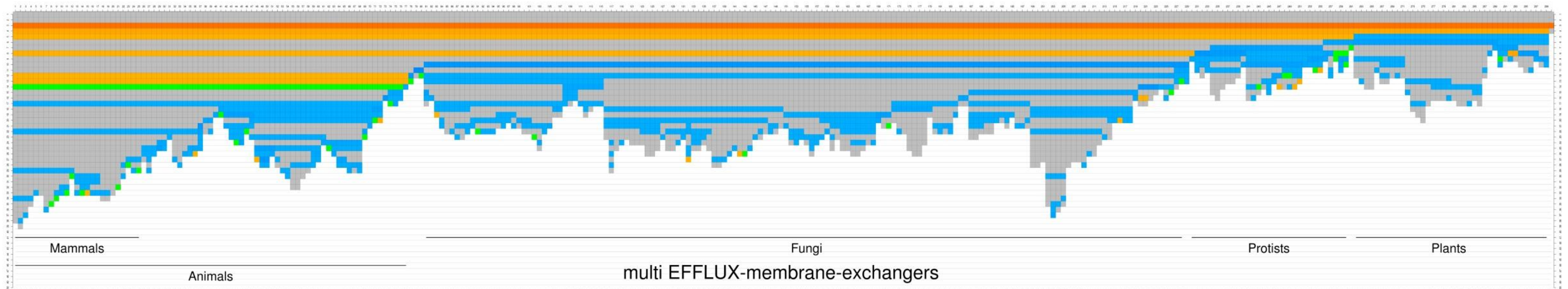

multi EFFLUX-membrane-exchangers

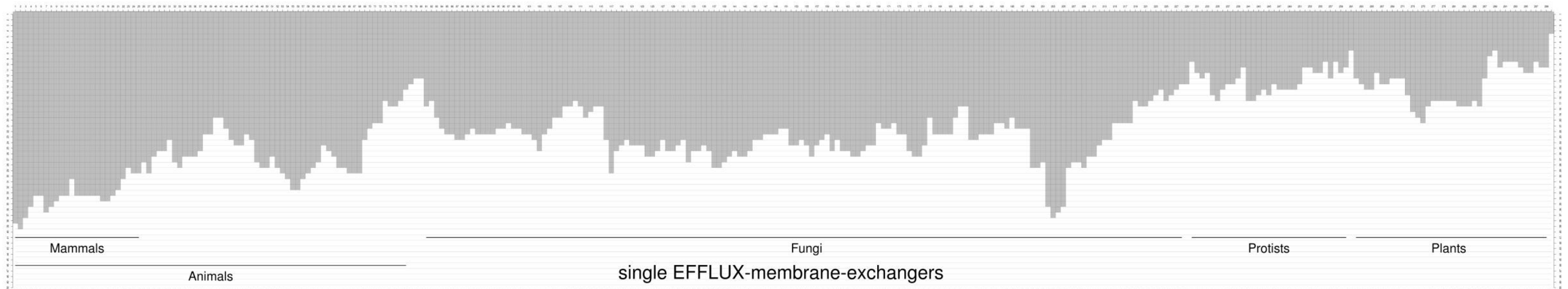

single EFFLUX-membrane-exchangers

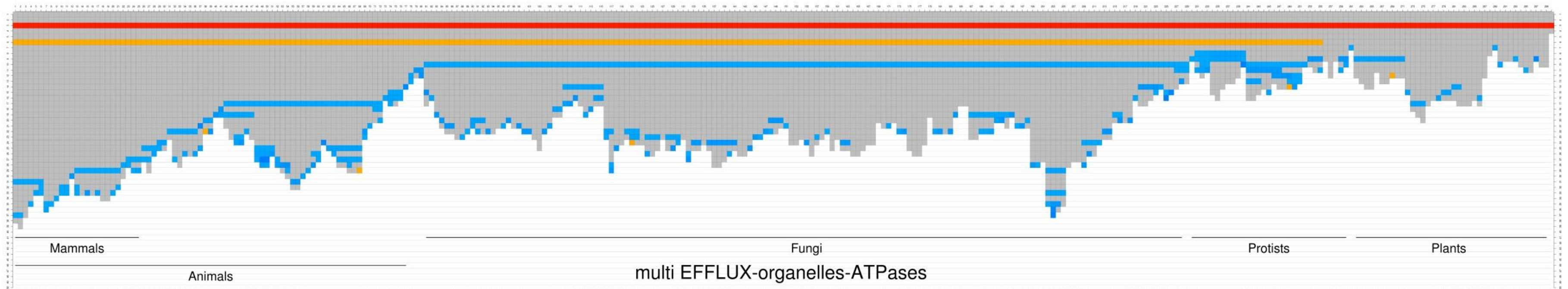

multi EFFLUX-organelles-ATPases

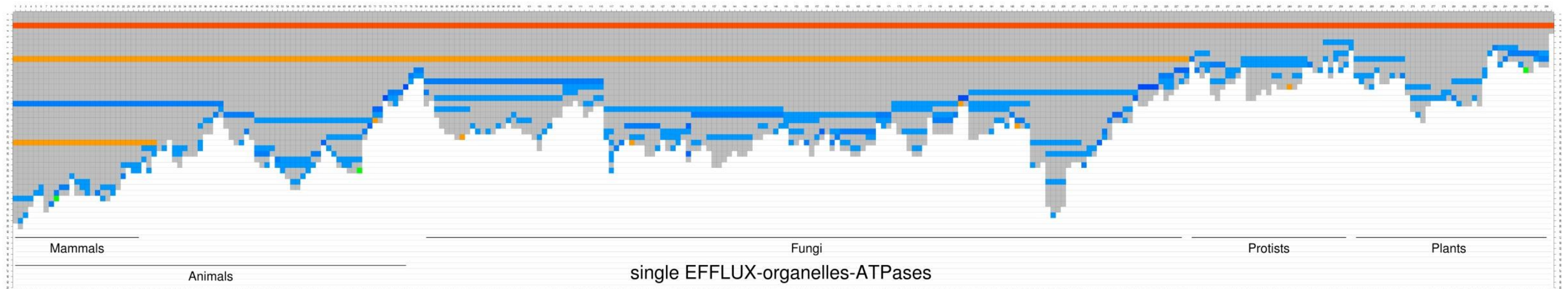

single EFFLUX-organelles-ATPases

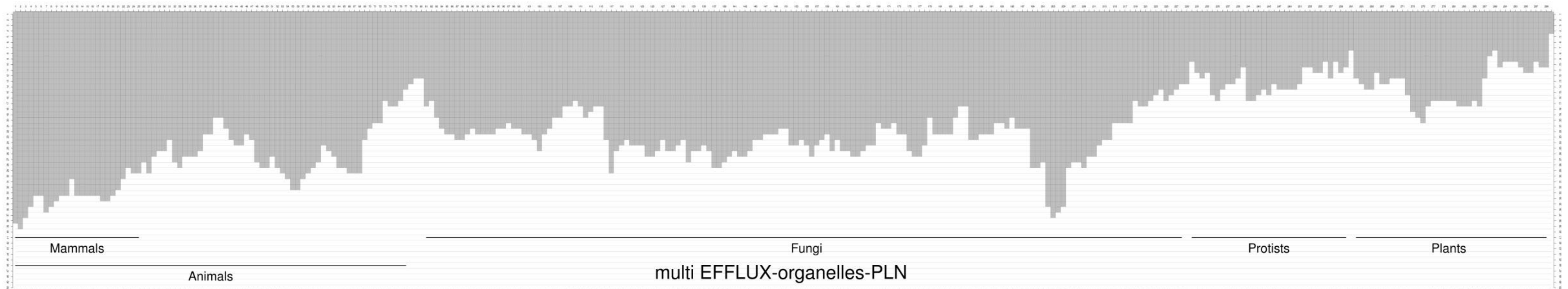

multi EFFLUX-organelles-PLN

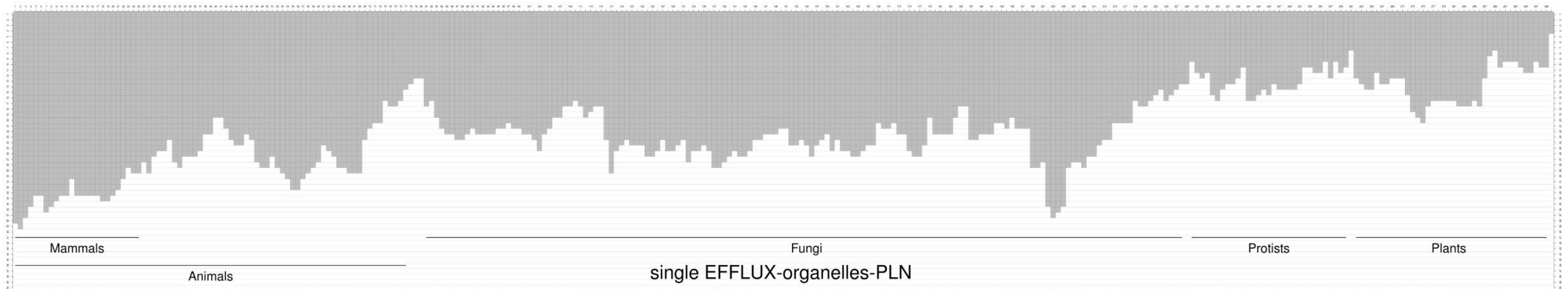

single EFFLUX-organelles-PLN

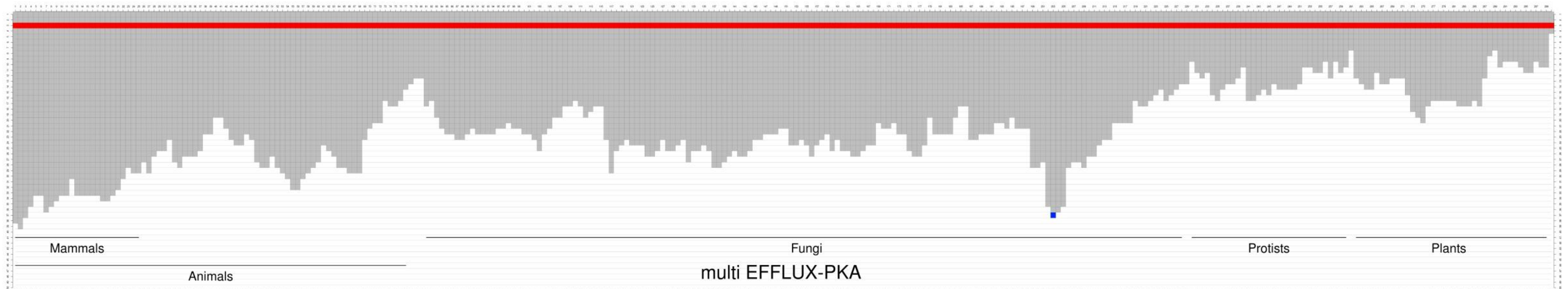

multi EFFLUX-PKA

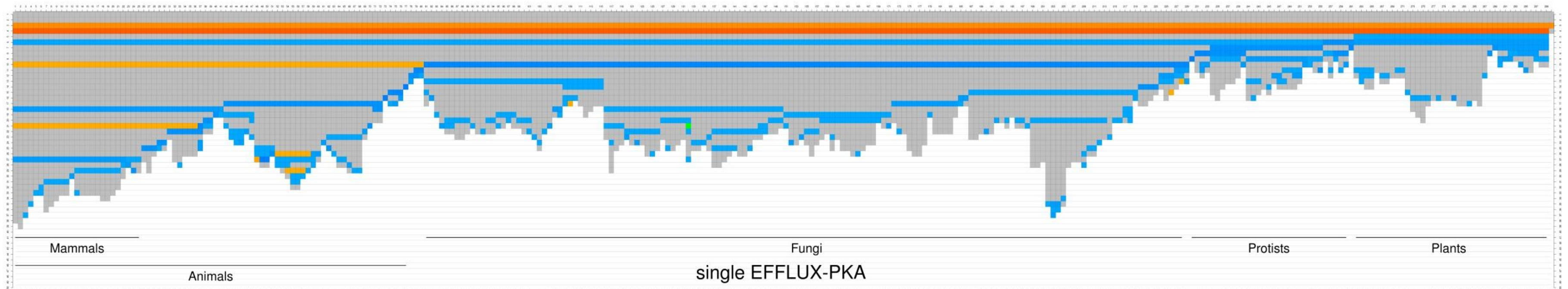

single EFFLUX-PKA

Decoding

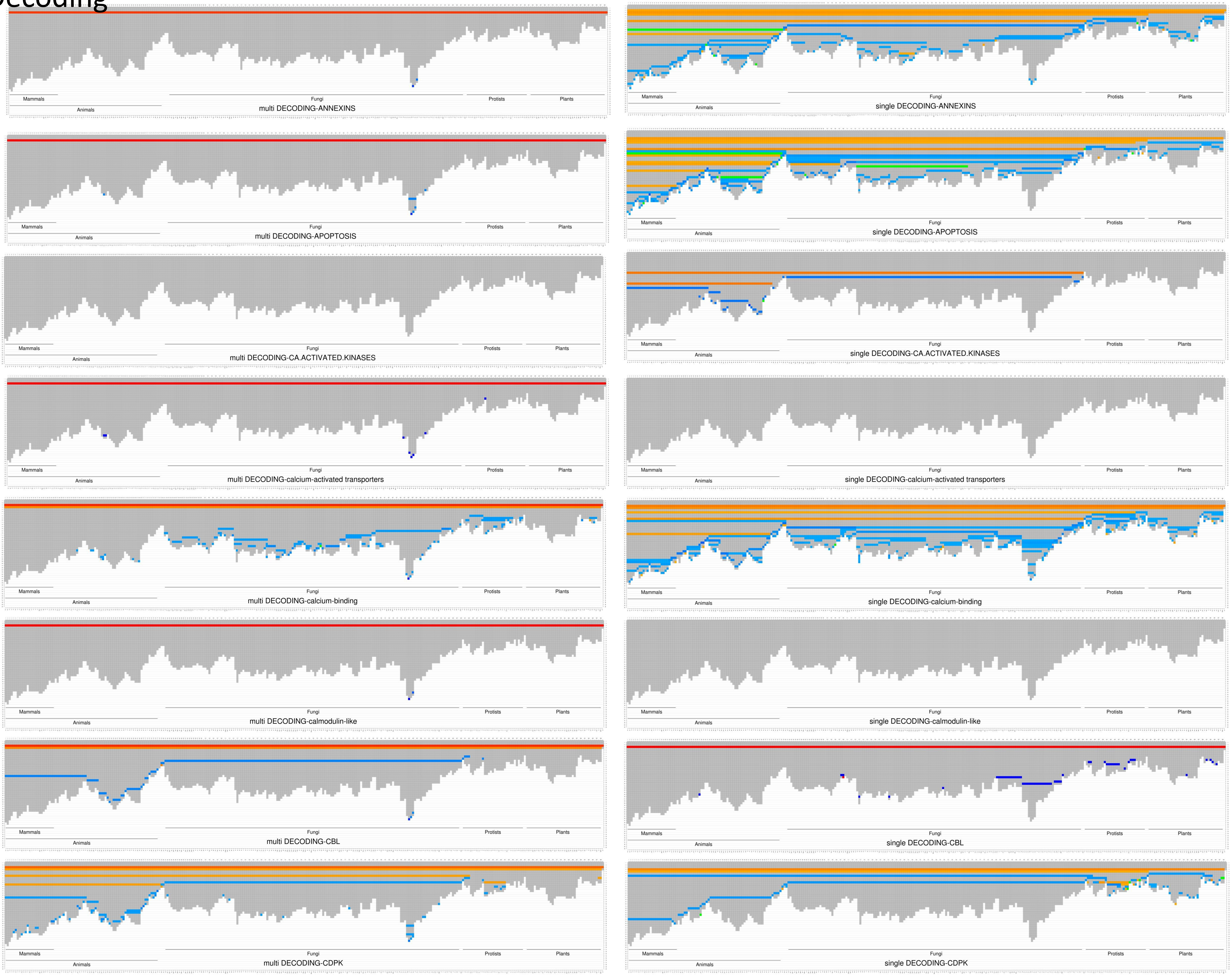

Mammals
Animals
Fungi
Protists
Plants
multi DECODING-ANNEXINS
single DECODING-ANNEXINS
multi DECODING-APOPTOSIS
single DECODING-APOPTOSIS
multi DECODING-CA.ACTIVATED.KINASES
single DECODING-CA.ACTIVATED.KINASES
multi DECODING-calcium-activated transporters
single DECODING-calcium-activated transporters
multi DECODING-calcium-binding
single DECODING-calcium-binding
multi DECODING-calmodulin-like
single DECODING-calmodulin-like
multi DECODING-CBL
single DECODING-CBL
multi DECODING-CDPK
single DECODING-CDPK

Decoding

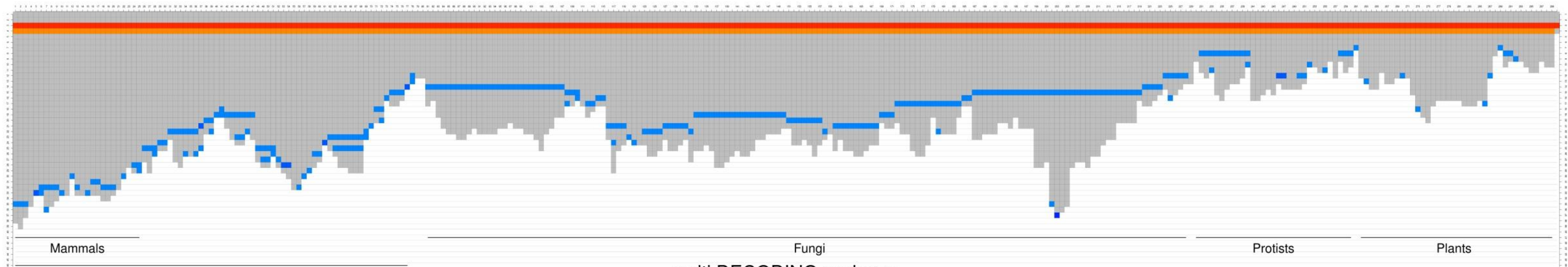

multi DECODING-cyclases

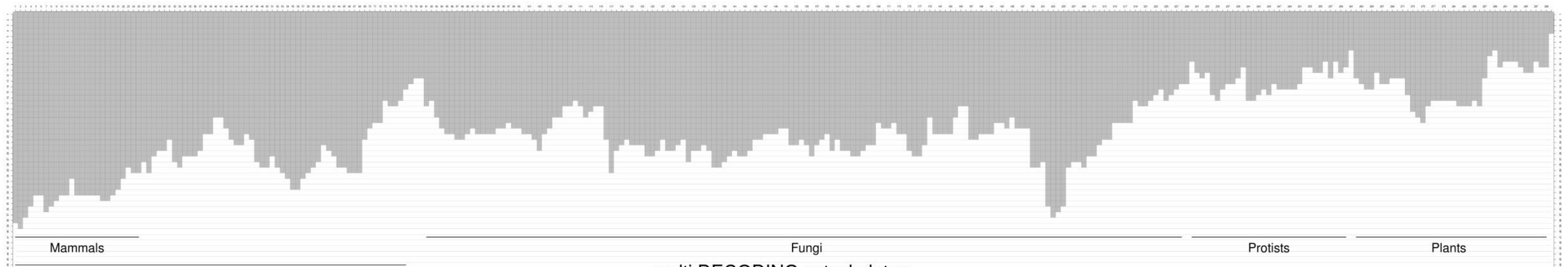

multi DECODING-cytoskeleton

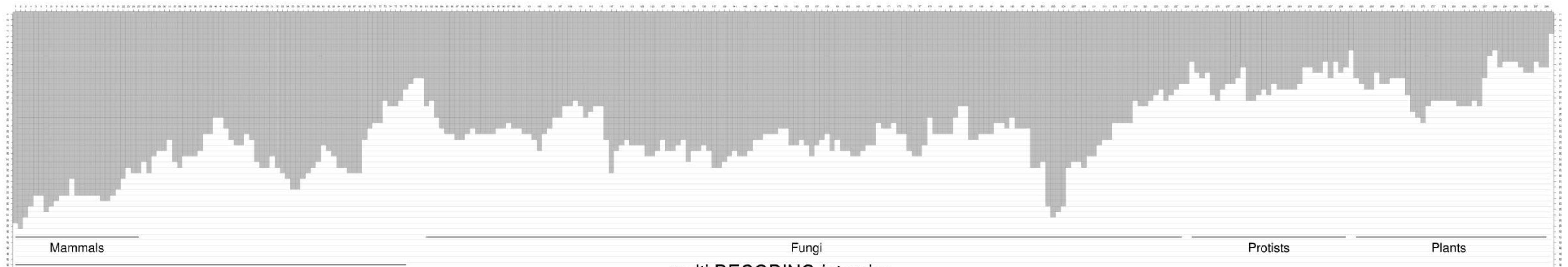

multi DECODING-integrins

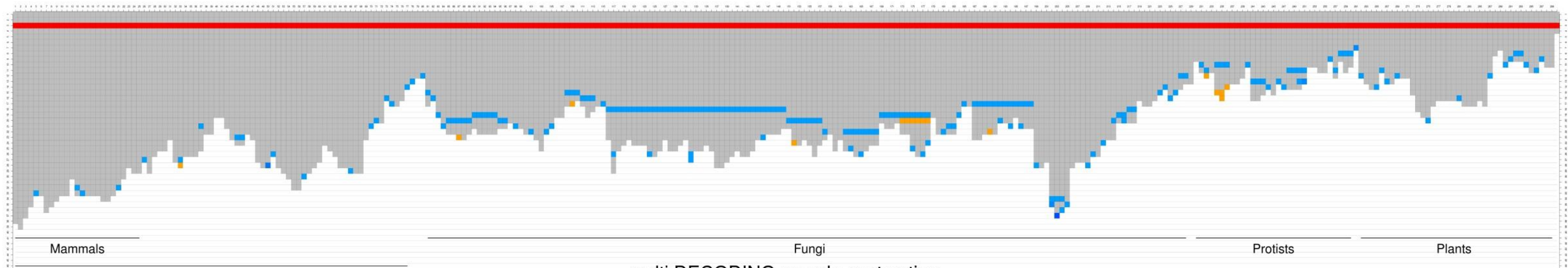

multi DECODING-muscle contraction

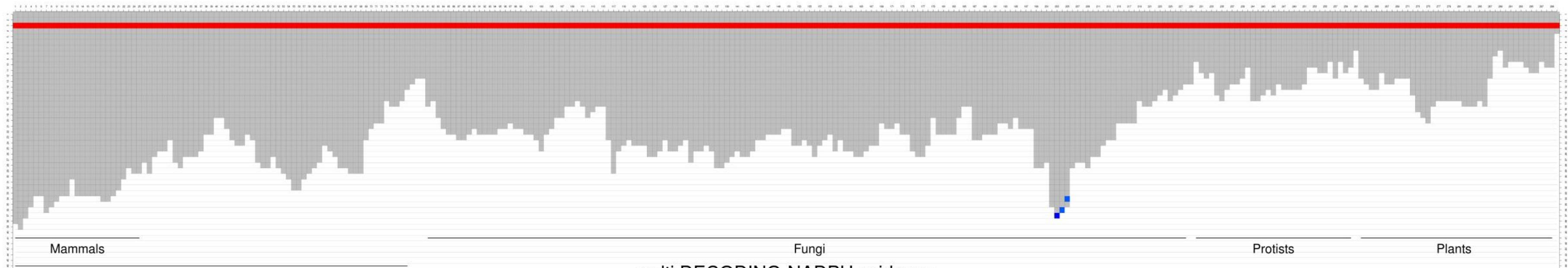

multi DECODING-NADPH oxidases

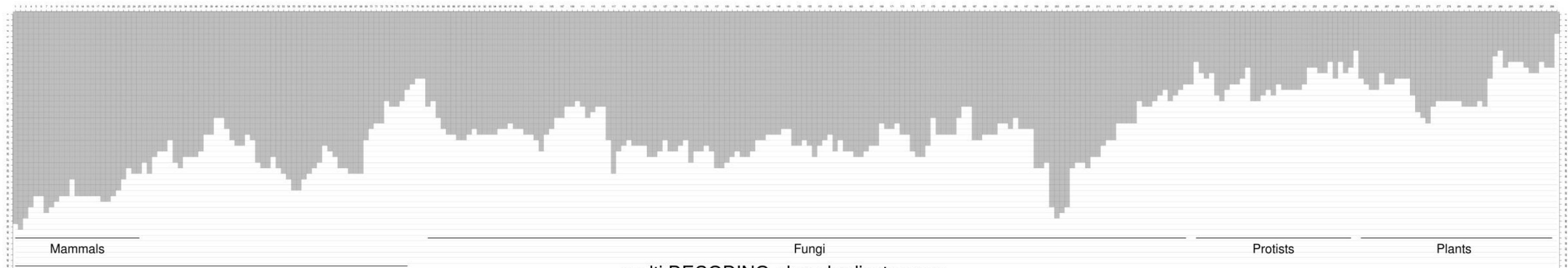

multi DECODING-phosphodiesterases

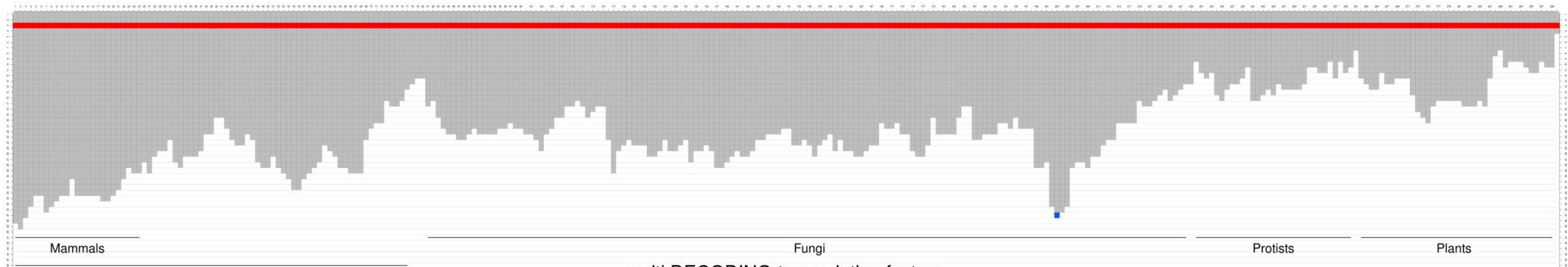

multi DECODING-transcription factors

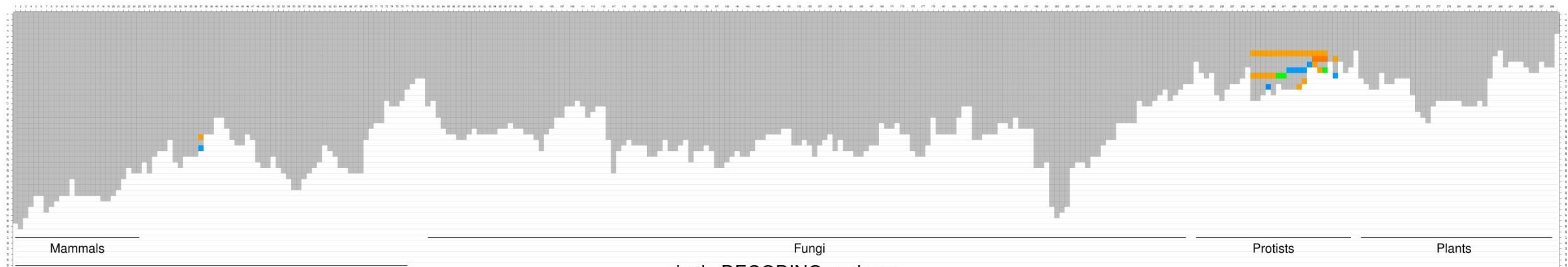

single DECODING-cyclases

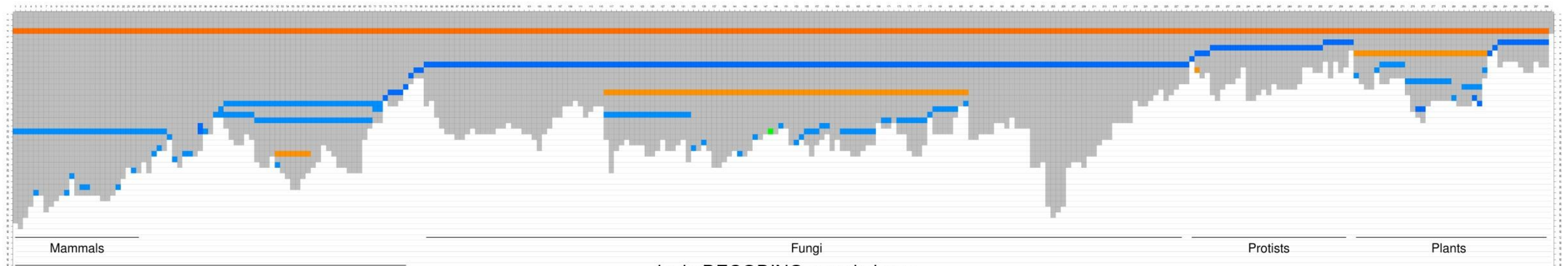

single DECODING-cytoskeleton

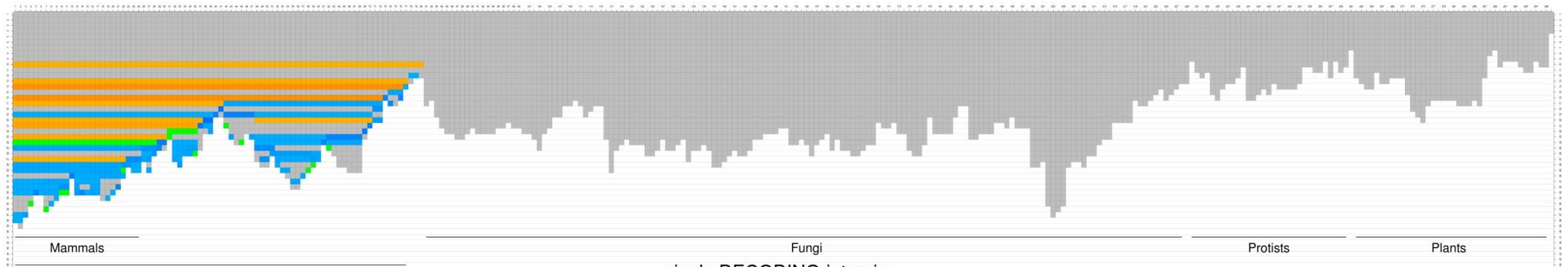

single DECODING-integrins

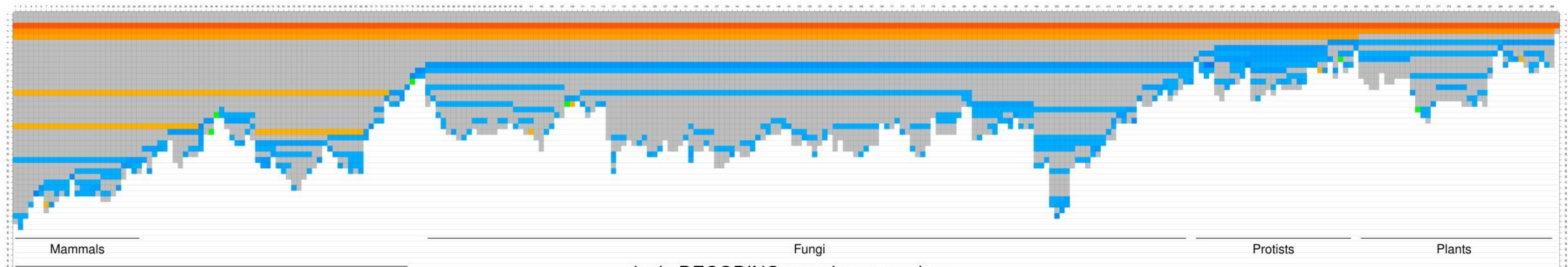

single DECODING-muscle contraction

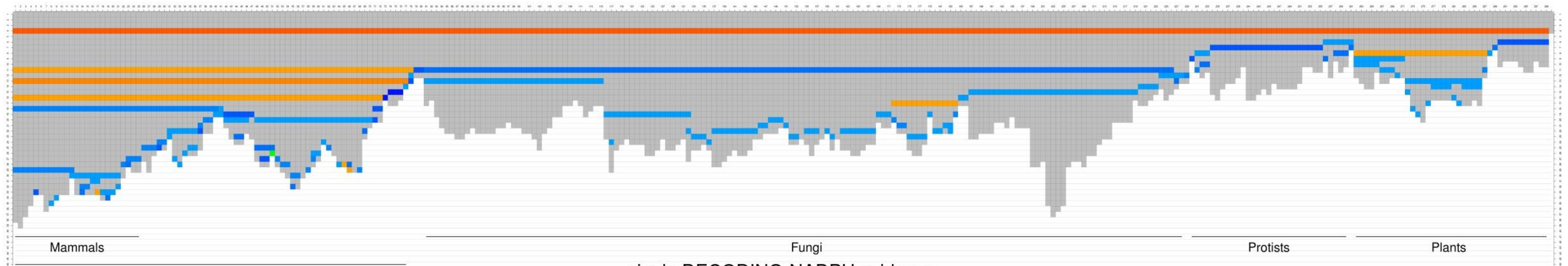

single DECODING-NADPH oxidases

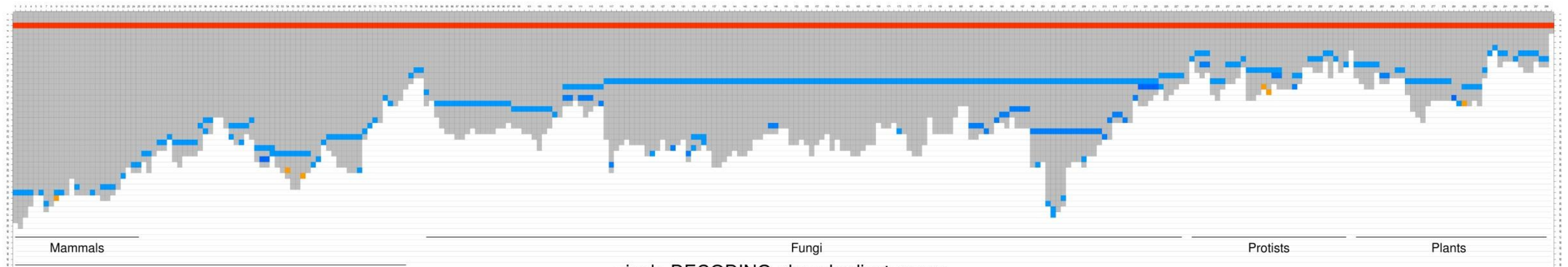

single DECODING-phosphodiesterases

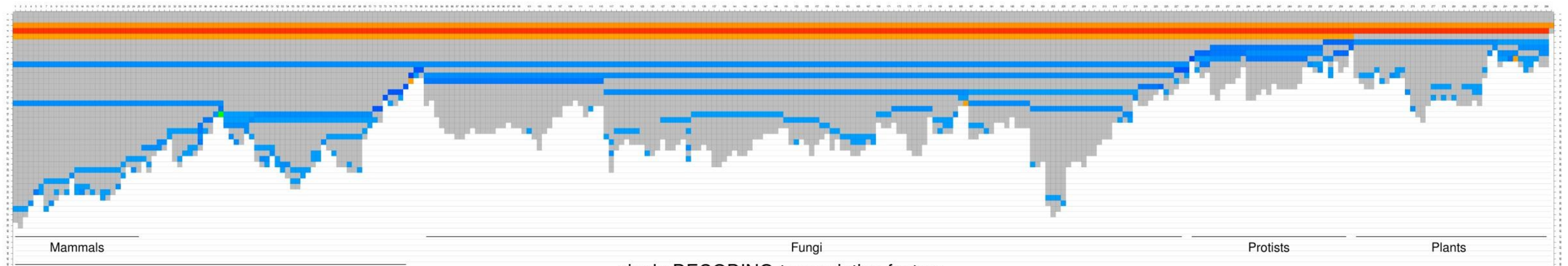

single DECODING-transcription factors

Relay

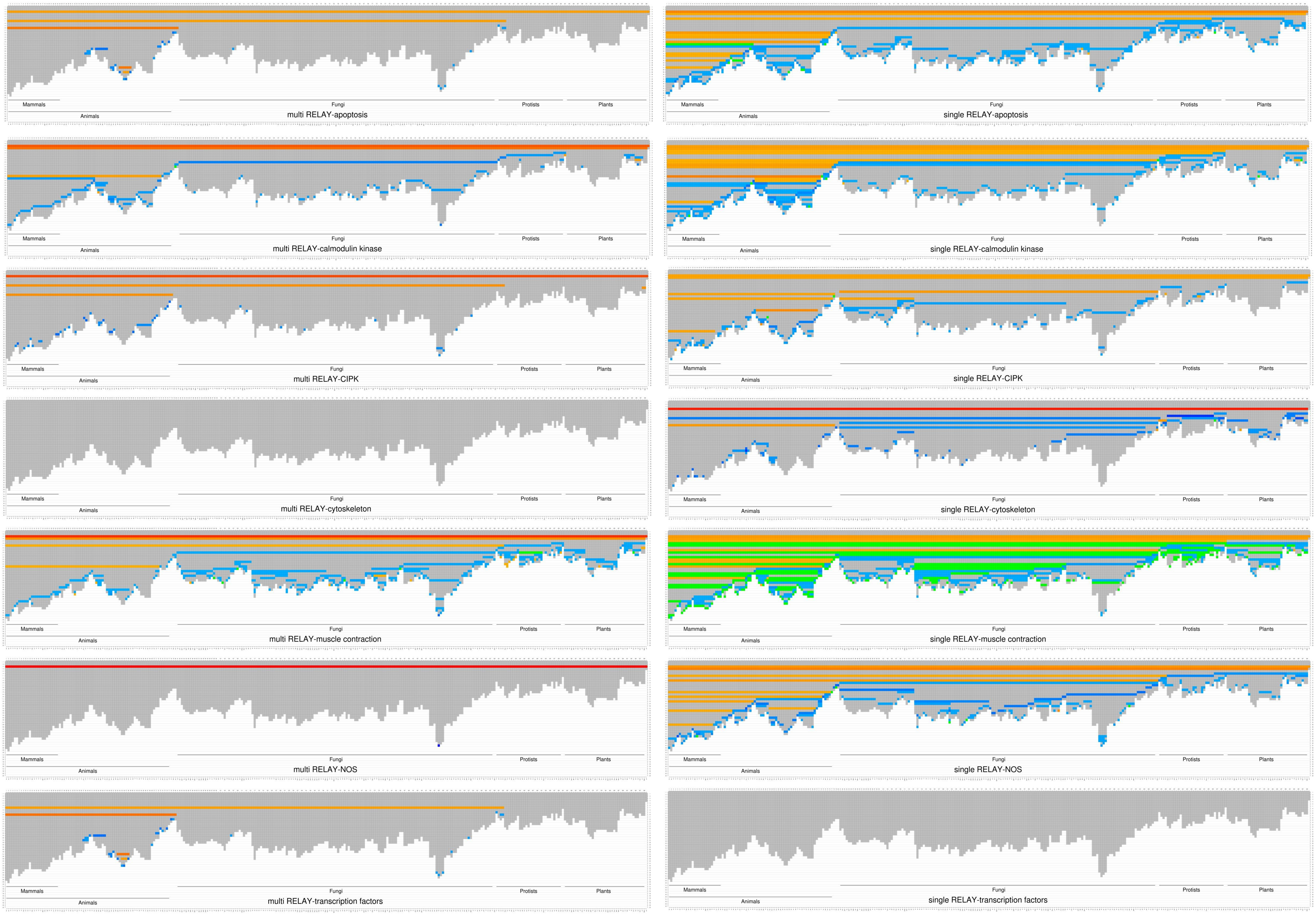
Mammals
Animals
Fungi
Protists
Plants
multi RELAY-apoptosis
single RELAY-apoptosis
multi RELAY-calmodulin kinase
single RELAY-calmodulin kinase
multi RELAY-CIPK
single RELAY-CIPK
multi RELAY-cytoskeleton
single RELAY-cytoskeleton
multi RELAY-muscle contraction
single RELAY-muscle contraction
multi RELAY-NOS
single RELAY-NOS
multi RELAY-transcription factors
single RELAY-transcription factors
